# Condensin targets and reduces unwound DNA structures associated with transcription in mitotic chromosome condensation

**DOI:** 10.1038/ncomms8815

**Published:** 2015-07-23

**Authors:** Takashi Sutani, Toyonori Sakata, Ryuichiro Nakato, Koji Masuda, Mai Ishibashi, Daisuke Yamashita, Yutaka Suzuki, Tatsuya Hirano, Masashige Bando, Katsuhiko Shirahige

**Affiliations:** 1 Research Center for Epigenetic Disease, Institute of Molecular and Cellular Biosciences, The University of Tokyo, Tokyo 113-0032, Japan; 2 Chromosome Dynamics Laboratory, RIKEN, Wako, Saitama 351-0198, Japan; 3 Department of Medical Genome Sciences, Graduate School of Frontier Sciences, The University of Tokyo, Kashiwa, Chiba 277-8561, Japan; 4 CREST, Japan Science and Technology Agency (JST), K's Gobancho 6F, 7, Gobancho, Chiyoda-ku, Tokyo 102-0076, Japan

## Abstract

Chromosome condensation is a hallmark of mitosis in eukaryotes and is a prerequisite for faithful segregation of genetic material to daughter cells. Here we show that condensin, which is essential for assembling condensed chromosomes, helps to preclude the detrimental effects of gene transcription on mitotic condensation. ChIP-seq profiling reveals that the fission yeast condensin preferentially binds to active protein-coding genes in a transcription-dependent manner during mitosis. Pharmacological and genetic attenuation of transcription largely rescue bulk chromosome segregation defects observed in condensin mutants. We also demonstrate that condensin is associated with and reduces unwound DNA segments generated by transcription, providing a direct link between an *in vitro* activity of condensin and its *in vivo* function. The human condensin isoform condensin I also binds to unwound DNA regions at the transcription start sites of active genes, implying that our findings uncover a fundamental feature of condensin complexes.

## Abstract (web summary)

Chromosome condensation is a prerequisite for faithful segregation of chromosomes to daughter cells. Here, the authors show that the condensin complex binds to protein-coding genes in a transcription-dependent manner during condensation, and reduces unwound DNA segments generated by transcription.

## Graphical abstract

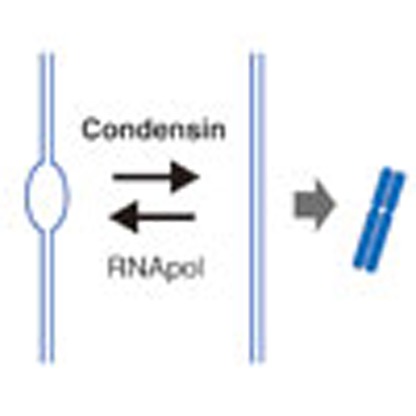

Each chromosomal DNA molecule in the interphase nucleus is disentangled from the others and organized into a compact rod-shaped structure on entry into mitosis. In many species, chromosome condensation requires two proteinaceous factors, DNA topoisomerase II and condensin^1^,^2^. Inactivation of either factor by a gene mutation or knockdown results in chromosome condensation defects and ultimately chromosome missegregation, demonstrating that mitotic condensation is a prerequisite for faithful segregation of chromosomes. Topoisomerase II is believed to disentangle DNA strands via its DNA strand passage function during the condensation process^3^. In contrast, despite extensive investigation, far less is understood about how condensin, a conserved five-subunit complex with ATPase domains, functions in this process^4^,^5^,^6^,^7^,^8^,^9^.

There are two prevailing models for the *in vivo* functions of condensin. One is that condensin functions as a crosslinker that provides a network of interactions between distant chromatin segments^7^,^8^. This model is largely derived from the structural similarity of condensin to its molecular cousin, cohesin, which makes a bridge between two sister chromatids^10^,^11^. The other is that condensin controls higher-order chromatin coiling to promote condensation^12^. The experimental basis for this model is the observation that human and frog condensin I, one of two condensin isoforms these species possess, can introduce positive supercoiling into a relaxed circular DNA in the presence of topoisomerase I *in vitro*^13^,^14^. To clarify how condensin acts on chromosomes, it is essential to know where in the genome condensin is bound and how it modulates configuration of chromosomal DNA in that region. Recent work^15^,^16^ has revealed an association of condensin I and II complexes with transcribed genes in chicken and *Caenorhabditis elegans* chromosomes, respectively, providing valuable insight into this question. It remains to be elucidated, however, how condensin functions at its binding sites.

The fission yeast *Schizosaccharomyces pombe* shows clear mitotic chromosome condensation despite its relatively small genome size^17^,^18^, making it an ideal system for investigating chromosome condensation in a genetically tractable organism. Previous work using DNA microarrays^19^ revealed enrichment of fission yeast condensin at transcribed loci, including transfer RNA (tRNA) and ribosomal protein genes, similar to the observations in chicken and *C. elegans*. Many mitotically active genes are not included^20^, however, in these detected binding sites, which limits the support for a link between transcription and condensin binding. Massively parallel sequencing has since become available and offers an opportunity to re-examine the distribution profile of condensin binding, as it enables more sensitive and accurate identification of DNA fragments.

In this study, we present improved, multiply validated, genome-wide profiles of condensin binding on fission yeast mitotic chromosomes. In addition, we demonstrate that these binding sites are associated with actively transcribed RNA polymerase II (RNAP2)-driven genes, and that at these sites transcription promotes and condensin prevents accumulation of unwound DNA structure, which is presumably detrimental to assembling properly condensed chromosomes.

## Results

### Genome-wide distribution of fission yeast condensin

**Figure 1:**
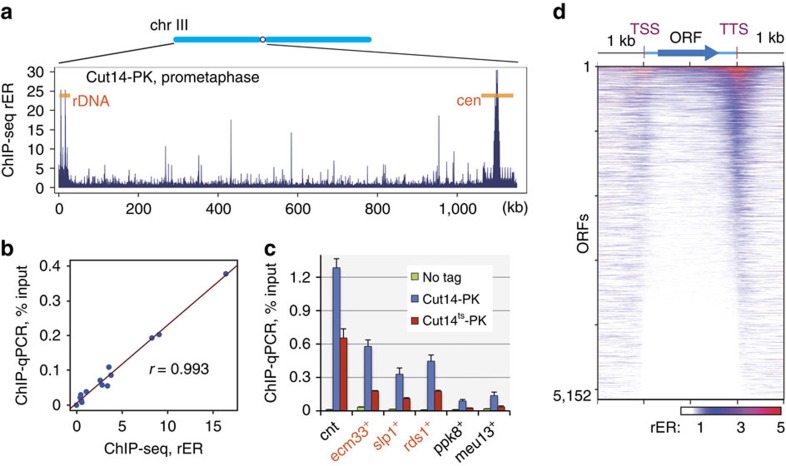
Identification of condensin binding sites in fission yeast mitotic chromosomes. (**a**) Binding profile of condensin complex along the chromosome III (chr III) left arm. Cells with an epitope-tagged condensin subunit (Cut14-PK) were arrested at prometaphase by the *nda3*-KM311 cold-sensitive tubulin allele and analysed by ChIP-seq using anti-PK antibody. The rER (vertical axis) reflects the binding probability at the corresponding genome site (horizontal axis); cen, centromere. (**b**) Verification of ChIP-seq results by qPCR. DNA isolated by Cut14-PK ChIP was measured by qPCR at 13 selected sites in the genome, including the identified condensin binding sites (Supplementary Fig. 1d). The resulting ChIP efficiency values (represented as % input) showed good correlation with ChIP-seq rERs, verifying the accuracy of ChIP-seq results. *r*, Pearson's correlation coefficient. (**c**) Physiological relevance of detected condensin binding. Chromosome binding of epitope-tagged wild-type and temperature-sensitive^35^ Cut14 proteins (Cut14-PK and Cut14^ts^-PK, respectively) was assayed by ChIP-qPCR. Cells were arrested in prometaphase by cultivating at 20 °C, a permissive temperature for *cut14-208* but restrictive temperature for the cold-sensitive *nda3* mutation. The mutant protein showed reduced binding at all locations tested, including the newly identified Cut14-enriched sites (orange), even at the permissive temperature. Each qPCR site is named after the nearby gene or genomic feature; cnt, central core regions of centromeres 1 and 3. Cut14^ts^ protein is not heat labile and is as stable as the wild-type protein^35^. No tag, untagged wild-type cells as a control. Error bars represent s.d. (*n*=2, technical replicates in qPCR). (**d**) Heat map of condensin enrichment for all protein-coding genes from 1 kb upstream of the TSS to 1 kb downstream of the TTS. Gene lengths are scaled to the same size. Genes are ranked from highest to lowest condensin enrichment.

*S. pombe* cells in which the condensin subunit Cut14 (*S. pombe* homologue of Smc2) was tagged with the PK epitope were arrested in prometaphase and subjected to chromatin immunoprecipitation sequencing (ChIP-seq) analysis^21^,^22^,^23^. The resulting profile (Fig. 1a) showed prominent peaks at centromeres and ribosomal DNA (rDNA) loci, corresponding to two major condensin binding sites identified in fission yeast^24^,^25^. In addition to these known loci, the profile revealed ∼340 novel sites with moderate but statistically significant condensin enrichment along the chromosome arms. Among these, 49 sites exhibited enrichment comparable to that seen at centromeres and rDNA loci (Supplementary Table 1). The validity of the profile was assessed through several means. The profile was reproducible and dependent on the presence of the epitope tag on condensin (Supplementary Fig. 1a−c). Moreover, quantitative PCR (qPCR) measurements at multiple genomic loci of ChIP-purified DNA from wild-type or condensin mutant cells verified that binding in the ChIP-seq profile was quantitatively accurate and relied on the functional integrity of the condensin complex (Fig. 1b,c and Supplementary Fig. 1d,e). We therefore conclude that the ChIP-seq profiles captured genuine condensin binding to chromosomes both precisely and accurately. It should be noted that these profiles do not indicate that condensin was absent from regions other than the detected peaks. Indeed, the results of both ChIP-seq and ChIP-qPCR show that a significant amount of condensin was found along the entire nuclear genome, although it was more prominent at the peaks (Supplementary Fig. 1d,f). Hereafter, we refer to the detected peaks as condensin binding sites and focus our analysis on these sites.

### Condensin localizes at active genes

**Figure 2:**
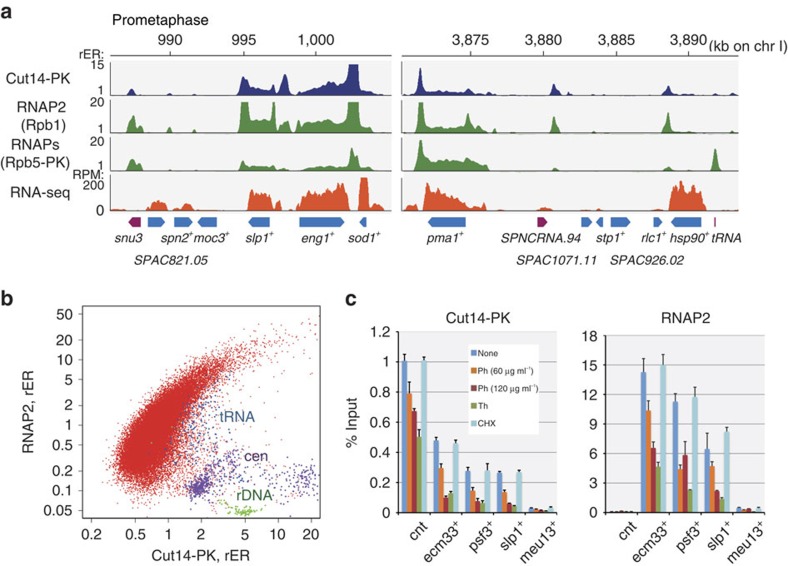
Transcription-dependent condensin binding at RNAP2-driven active genes. (**a**) ChIP-seq profiles of condensin (Cut14-PK), RNAP2 (detected by monoclonal antibody 8WG16, recognizing the largest subunit Rpb1) and all RNA polymerases (RNAPs, detected using the PK epitope on the common subunit Rpb5), as well as a poly(A)-selected RNA-seq profile. All profiles are from prometaphase cells. Annotated ORFs (cyan) and other transcripts (non-coding RNA and tRNA, magenta) are shown at the bottom. chr I, chromosome I. (**b**) Genome-wide correlation plot of Cut14-PK and RNAP2 ChIP-seq results. Purple, green and blue correspond to centromeres (cen), rDNA and tRNA gene loci, respectively. (**c**) Transcription-dependent condensin binding. Cut14-PK cells in prometaphase were treated with the transcription inhibitor 1,10-phenanthroline (Ph; 60 or 120 μg ml^−1^) or thiolutin (Th; 20 μg ml^−1^) for 30 min and analysed by anti-PK or anti-RNAP2 ChIP-qPCR. The translation inhibitor cycloheximide (CHX; 100 μg ml^-1^) was used as a negative control. Error bars represent s.d. (*n*=2, technical replicates in qPCR). cnt, central core regions of centromeres 1 and 3.

ChIP-seq profiles indicated that condensin was enriched on a subset of genes, with notable accumulation around transcription termination sites (TTSs; Fig. 1d). Genes that show expression peaks in the M–G1 phases^20^ were significantly enriched for condensin binding (for example, *ecm33*^+^, *eng1*^+^ *cdc22*^+^; *P*=3.3 × 10^–11^; Fisher's exact test), indicating that condensin was localized on mitotically transcribed genes. Indeed, ChIP-seq analysis of RNAP2 revealed that condensin was perfectly co-localized with RNAP2 in mitotic cells (Fig. 2a). With the exception of centromere and rDNA regions, the co-localization was verified in a genome-wide correlation plot (Fig. 2b). Moreover, the RNAP2 binding detected by ChIP-seq reflected actively transcribing polymerase, because RNA-seq analysis revealed the presence of mRNAs of the corresponding genes (Fig. 2a). Reverse transcription–qPCR (RT–qPCR) and immunostaining for active RNAP2 also confirmed the presence of active transcription in fission yeast mitotic cells (Supplementary Fig. 2a,b). Consistent with previous work using the ChIP-chip method^19^, the current analysis also detected moderate accumulation of condensin at many tRNA genes (Fig. 2b). The current ChIP-seq analysis therefore successfully extended the previous study, providing condensin distribution profiles with increased sensitivity and accuracy.

We next examined whether condensin binding to RNAP2-driven transcriptionally active genes, the major binding sites along the chromosome arms, was dependent on transcription. Treatment with the RNA polymerase inhibitors 1,10-phenanthroline or thiolutin^26^,^27^ reduced the amount of both RNAP2 and condensin associated with condensin binding sites (Fig. 2c). In contrast, the translation inhibitor cycloheximide had no effect, precluding the possibility that an attenuation of protein synthesis caused dissociation of condensin. Our genome-wide correlation plot suggests that 1,10-phenanthroline treatment caused condensin to dissociate from chromosomes, not translocate along them (Supplementary Fig. 2c). Taken together, these results indicate that condensin binding to chromosomes depends on transcription. Time course analysis of 1,10-phenanthroline treatment further revealed that dissociation of condensin occurred significantly more slowly than that of RNAP2 (Supplementary Fig. 2d), implying that condensin recognizes not RNAP2 itself, but something else coupled with transcriptional activity (such as a locally unwound DNA structure, see below). An additional experiment in which expression of a foreign gene was induced by a mitotically active promoter also supported the proposition that active transcription is sufficient for condensin recruitment (Supplementary Fig. 2e,f).

### Transcription attenuation rescues condensin mutants

**Figure 3:**
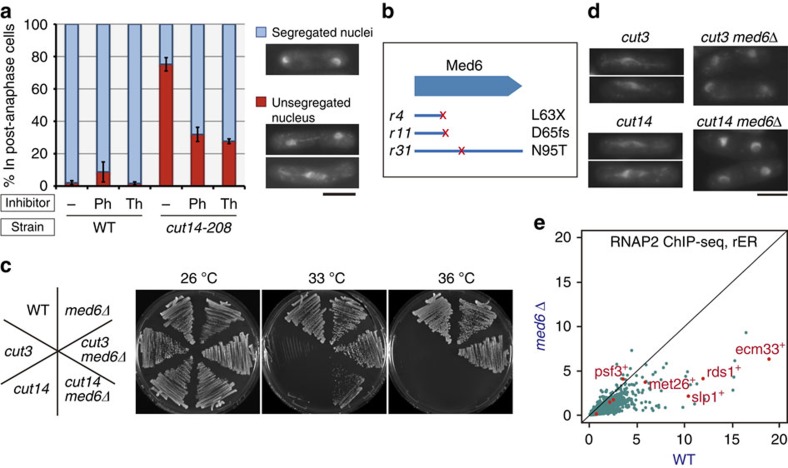
Suppression of chromosome segregation defect in condensin mutants by transcriptional attenuation. (**a**) Wild-type (WT) or temperature-sensitive condensin mutant (*cut14-208*) cells were arrested at prometaphase, treated with or without a transcription inhibitor (1,10-phenanthroline (Ph; 60 μg ml^−1^) or thiolutin (Th; 1 μg ml^−1^)) for 30 min and then released from arrest at the restrictive temperature (34 °C) for 15 min. Cells were fixed, and the nuclear morphology in post-anaphase cells was examined by DAPI staining. The frequency of stretched but unsegregated nuclei in *cut14-208* cells was significantly reduced by inhibitor treatment. Error bars represent s.e.m. (*n*=3, independent experiments). Scale bar, 5 μm. (**b**) Suppressor mutations in three independent phenotypic revertants of *cut3* were revealed by whole-genome sequencing to reside in *med6* (Mediator complex subunit 6). X, nonsense mutation; fs, frameshift mutation. (**c**) Rescue of growth defect in condensin mutants by *med6* deletion (*med6*Δ). Med6 depletion rescued *cut3-477* completely at 36 °C and partially suppressed the more severe *cut14-208* at 33 °C. (**d**) Rescue of chromosome segregation defect in condensin mutants by *med6*Δ. Cells were cultivated at restrictive temperatures (36 °C, 4 h for *cut3*, *cut3 med6*Δ; 34 °C, 2 h for *cut14*, *cut14 med6*Δ) and then fixed. Nuclear morphology in mitotic cells was revealed by DAPI staining. Scale bar, 5 μm. (**e**) Genome-wide comparison of RNAP2 binding between WT and *med6*Δ cells. The amount of RNAP2 bound to each protein-coding gene was calculated based on ChIP-seq and ChIP-qPCR results and plotted. RNAP2 binding was decreased at most genes in *med6*Δ. Genes monitored by qPCR (Supplementary Fig. 3b) are shown in red.

A key question that arises from these analyses is why condensin localizes at transcriptionally active genes during mitosis. One possibility is that active genes act as footholds, or *cis*-acting DNA elements, from which condensin promotes global chromosome condensation. In this model, condensin dissociation caused by transcription inhibition should cause condensation defects, eventually leading to chromosome missegregation, as seen in condensin mutants^18^,^28^. Treatment of wild-type mitotic cells with transcription inhibitors, however, revealed little or no increase in missegregation (Fig. 3a). An alternative model is that actively transcribed regions require a higher level of condensin activity to be assembled into condensed chromosomes. To test this idea, cells of *cut14-208*, a temperature-sensitive condensin subunit mutant^18^, were arrested at prometaphase and then released into the cell cycle at a restrictive temperature (34 °C) in the absence or presence of an RNA polymerase inhibitor (1,10-phenanthroline or thiolutin). Untreated *cut14* cells showed a condensation defect leading to a severe chromosome segregation defect at the restrictive temperature. Addition of either RNA polymerase inhibitor markedly rescued the segregation defect (Fig. 3a), supporting the second model. Moreover, these same transcription inhibitors partially rescued the temperature-sensitive growth of condensin mutants but did not suppress that of the DNA topoisomerase II mutant *top2* (ref. 29), another factor that functions in chromosome condensation (Supplementary Fig. 3a), indicating that growth rescue by transcription inhibition is specific to condensin mutants. We therefore conclude that active transcription on mitotic chromosomes impedes chromosome segregation when condensin function is compromised.

The idea that transcription is detrimental to condensation is further supported by newly identified genetic interactions. When cells of *cut3-477*, a temperature-sensitive mutant of the Cut3 (*S. pombe* homologue of Smc4) condensin subunit^18^, were grown at a restrictive temperature (34 °C), phenotypic revertants appeared and formed colonies at a low frequency (typically ∼1 in 10^7^ cells). These revertants are likely to carry spontaneous suppressor mutations. Whole-genome sequencing^30^ revealed that three independent revertants possessed mutations in the same open reading frame (ORF), *med6* (also known as *pmc5*; Fig. 3b). The identified mutations include nonsense and frameshift mutations, suggesting that loss of Med6 function suppresses the *cut3* mutant. Indeed, Med6 is not essential for cell survival^31^, and *med6* gene deletion fully restored the ability of *cut3* mutant cells to form colonies and execute faithful chromosome segregation at the restrictive temperature of 36 °C (Fig. 3c,d). The *med6* deletion also partially rescued the *cut14-208* mutant at a lower temperature (Fig. 3c,d). Med6 is a subunit of the Mediator complex, which constitutes the basic transcriptional apparatus (including general transcription factors and RNAP2) and is required for activator-dependent, RNAP2-driven transcription^32^,^33^. *med6* deletion is therefore expected to reduce transcription for at least a subset of protein-coding genes. Thus, we performed RNAP2 ChIP-qPCR in prometaphase-arrested *med6*-deleted cells (*med6*Δ). Binding of RNAP2 was reduced at most sites measured (Supplementary Fig. 3b). Quantitative comparison of ChIP-seq data sets between wild-type and *med6*Δ cells further revealed that *med6* deletion caused a marked reduction in transcription of the majority of RNAP2-dependent genes on a genome-wide scale (Fig. 3e). Taken together, these analyses demonstrate that intact condensin function is required for proper condensation and faithful segregation of mitotic chromosomes with active RNAP2-driven transcription. The selective binding of condensin to active genes likely reflects a higher demand for its activity to accommodate these regions into condensed chromatin.

### Condensin binds to unwound DNA

To understand how condensin functions at highly transcribed genes, we first examined whether condensin negatively regulates transcription in mitotic cells. We created a fission yeast strain in which Cnd2 (*S. pombe* homologue of NCAPH), an essential subunit of condensin^28^, can be depleted by shutting off its expression (Supplementary Fig. 4a,b). This allowed us to eliminate active condensin almost completely when cells were arrested in prometaphase (Supplementary Fig. 4c–e). By RT–qPCR, we found that in Cnd2-depleted (Cnd2(−)) cells, the transcript levels of genes with condensin binding was not increased, but rather was slightly decreased (Supplementary Fig. 5a). In addition, immunoblotting revealed that the amount of active RNAP2 (phosphorylated at Ser2 or Ser5 in the C-terminal domain repeats^34^) was almost indistinguishable between wild-type and Cnd2(−) cells (Supplementary Fig. 5b). Thus, condensin did not actively repress transcription in fission yeast mitotic cells. We also investigated whether condensin acts by regulating chromatin configuration at active genes. Using histone H3 ChIP-seq, we found that nucleosome distribution was almost identical between wild-type and Cnd2(−) cells (Supplementary Fig. 5c), indicating that this is not the case.

**Figure 4:**
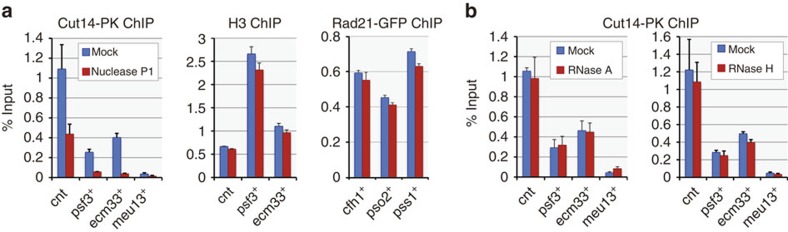
Presence of ssDNA at condensin binding sites. (**a**) Treatment of condensin-bound DNA fragments with nuclease P1, which is specific to ssDNA/single-stranded RNA. DNA fragments purified by Cut14-PK ChIP from prometaphase cells were treated with P1 on beads and then eluted and measured by qPCR (left). P1 sensitivity was specific to condensin-bound fragments, because bulk DNA at the same sites (purified by anti-histone H3 ChIP from prometaphase cells) or cohesin-associated DNA (purified by Rad21-GFP ChIP from asynchronous cells) showed no sensitivity (middle and right, respectively). (**b**) RNase treatment of condensin-bound DNA fragments. RNase A or RNase H treatment, which digests single-stranded RNA or RNA within DNA:RNA hybrids, respectively, caused no reduction in qPCR measurements, precluding the possibility that the condensin-DNA association is mediated by RNA. Error bars represent s.d. (*n*=2, technical replicates in qPCR). cnt, central core regions of centromeres 1 and 3.

The Cut3-Cut14 heterodimer, the core component of the condensin complex, promotes renaturation of complementary single-stranded DNA (ssDNA) fragments and has a higher affinity for ssDNA than double-stranded DNA *in vitro*^35^,^36^. Because transcription is accompanied by DNA unwinding^37^, we speculated that unwound DNA segments in transcribed genes might be targeted and restored by condensin. We tested this idea first by examining whether condensin-bound DNA contains single-stranded regions. DNA associated with condensin was isolated by ChIP, treated on the immunoprecipitation beads with nuclease P1 (which digests ssDNA or single-stranded RNA) and then eluted from the beads. qPCR quantification revealed that the majority of condensin-bound DNA is sensitive to P1 (Fig. 4a). This sensitivity to nuclease P1 was specific to condensin-bound DNA, because DNA associated with the related complex cohesin^4^,^10^ (purified by Rad21-GFP ChIP) showed no sensitivity to nuclease P1 (Fig. 4a). Bulk nucleosomal DNA purified by histone H3 ChIP also showed no P1 sensitivity at condensin binding sites (Fig. 4a), revealing that condensin binding was directly linked to P1-sensitive structures. Finally, treatment with RNases instead of nuclease P1 produced no reduction in the amount of recovered DNA (Fig. 4b), suggesting that P1 sensitivity was not due to digestion of RNA. Taken together, these results indicate that condensin-bound DNA contains ssDNA.

**Figure 5:**
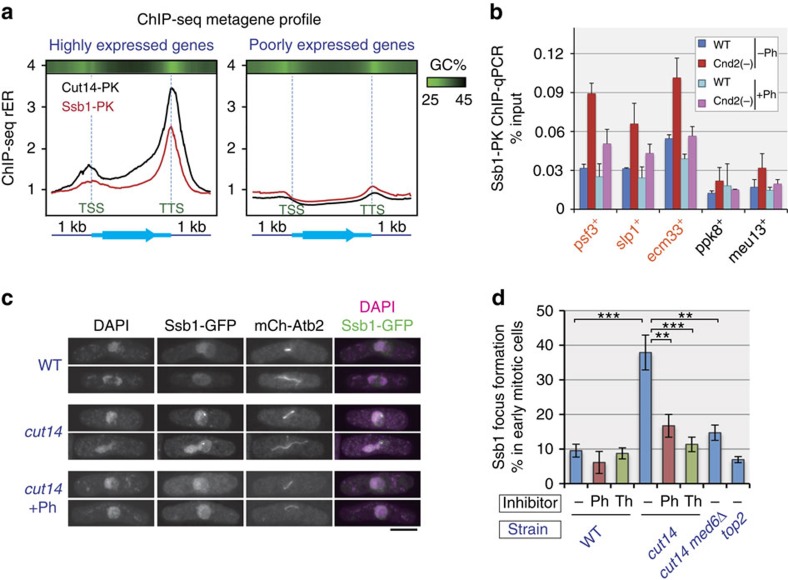
Opposing roles of transcription and condensin in DNA unwinding. (**a**) Metagene ChIP-seq profiles of condensin (Cut14-PK) and the eukaryotic ssDNA-binding factor RPA (Ssb1-PK). Distribution profiles were averaged over highly expressed genes in prometaphase cells (top 10%) as well as poorly expressed genes (bottom 50%). The profile is from 1 kb upstream of the TSS to 1 kb downstream of the TTS, and gene lengths were scaled to the same size. Top bars represent average GC content in a 100-bp window (bright green indicates lower GC content). Ssb1 is co-localized with condensin around the TTS of highly expressed genes. (**b**) ChIP-qPCR analysis of Ssb1-PK in prometaphase-arrested wild-type and condensin-depleted cells. Condensin depletion increased chromosomal binding of Ssb1 at condensin binding sites (orange), but this effect was attenuated by the transcription inhibitor 1,10-phenanthroline (Ph). Error bars represent s.d. (*n*=2, technical replicates in qPCR). (**c**,**d**) Wild-type (WT), *cut14*, *cut14 med6*Δ and *top2* cells expressing GFP-tagged Ssb1 and mCherry-tagged Atb2 (encoding α-tubulin) were cultured at 34 °C for 1 h and then fixed. Ph and thiolutin (Th) indicate treatment for the last 30 min with Ph (120 μg ml^−1^) and Th (2 μg ml^−1^), respectively. Micrographs of representative mitotic cells (**c**) and frequency of Ssb1 focus formation in nuclei of early mitotic cells with short spindles (**d**) are shown. Untreated *cut14* cells showed elevated levels of Ssb1 focus formation compared with WT, drug-treated *cut14* or *cut14 med6*Δ cells (**P<0.01 and ***P<0.001; Welch's *t*-test, one tailed), indicating that transcription promotes and condensin represses the accumulation of unwound DNA segments in mitotic cells. Scale bar, 5 μm. Error bars represent s.e.m. (*n*=7 independent experiments for *cut14*, *n*=4 for *cut14*+Ph, *n*=3 for others).

Binding of condensin to unwound DNA was further supported by ChIP-seq analysis of Ssb1, the largest subunit of replication protein A (RPA). Because RPA is a eukaryotic ssDNA-binding factor^38^, Ssb1 localization demonstrates the presence of ssDNA regions. Similar to condensin, Ssb1 was enriched on highly expressed genes, with significant accumulation around the TTS, and little to no Ssb1 was seen on unexpressed genes (Fig. 5a). It is noteworthy that DNA around the TTS is relatively guanine-cytosine (GC) poor, which could explain why the unwound DNA structure produced by transcription resists rewinding to double-stranded DNA. Collectively, these results strongly demonstrate that condensin on mitotic chromosomes is bound to the unwound DNA in transcribed genes.

### Restoration of unwound DNA by condensin

We next examined how condensin modulates unwound DNA *in vivo*. We measured the amount of chromosome-bound Ssb1 in wild-type cells by ChIP-qPCR and confirmed that Ssb1 binds preferentially at condensin binding sites over other sites. In Cnd2(−) cells, the amount of bound Ssb1 was increased ∼2-fold, and this effect was attenuated by 1,10-phenanthroline treatment (Fig. 5b). These results are consistent with the idea that condensin rewinds DNA at transcriptionally active sites via its DNA renaturation activity.

This notion was further assessed by cytological approaches. Previously, Akai *et al*.^39^ reported that yellow fluorescent protein (YFP)-tagged Ssb1 forms foci within nuclei of condensin mutant cells during mitosis. The observed Ssb1-YFP foci presumably indicate the presence of long RPA-coated stretches of ssDNA, which are possibly clustered in the nucleus. We conducted a similar analysis and confirmed that *cut14-208* condensin mutant cells formed Ssb1-GFP (green fluorescent protein) foci more frequently (∼40%) than wild-type cells (∼10%) during early mitosis (Fig. 5c,d). Condensin has an additional role in DNA damage repair^40^, and its impairment may lead to increased formation of Ssb1 foci. Alternatively, the chromosome stretching observed in anaphase nuclei of condensin mutant cells might be a direct cause of Ssb1 focus formation. Several lines of evidence preclude both of these possibilities: (i) the condensin mutant allele used in our analysis possesses an intact interphase function in DNA damage repair^40^; (ii) focus counting was conducted in early mitotic cells possessing short spindles, indicating that the nuclei were free of both DNA damage (which the DNA damage checkpoint can sense) and chromosome stretching imposed by spindle elongation; (iii) an elevated level of Ssb1 focus formation was also observed when *cut1*4 mutant cells were first arrested in prometaphase and then transferred to the restrictive temperature for the condensin mutant (Supplementary Fig. 6); and (iv) the *top2* mutant, which also shows chromosome stretching during anaphase^29^, demonstrated no increase in Ssb1 focus formation (Fig. 5d). We therefore concluded that the increased appearance of Ssb1 foci is directly linked to the defect in a role of condensin during mitosis.

We then examined whether the elevated formation of Ssb1 foci in *cut14* mutant cells is dependent on transcription. When the cells were treated with 1,10-phenanthroline or thiolutin, the frequency of *cut14* cells with Ssb1 foci was greatly reduced (Fig. 5c,d). In addition, *med6*Δ suppressed the formation of Ssb1 foci (Fig. 5d). Taken together, these data are consistent with the above-mentioned ChIP-seq/ChIP-qPCR results and strongly suggest that DNA that has been unwound by transcription in mitotic chromosomes is restored by the activity of the condensin complex.

### Binding of human condensin I to active genes

**Figure 6:**
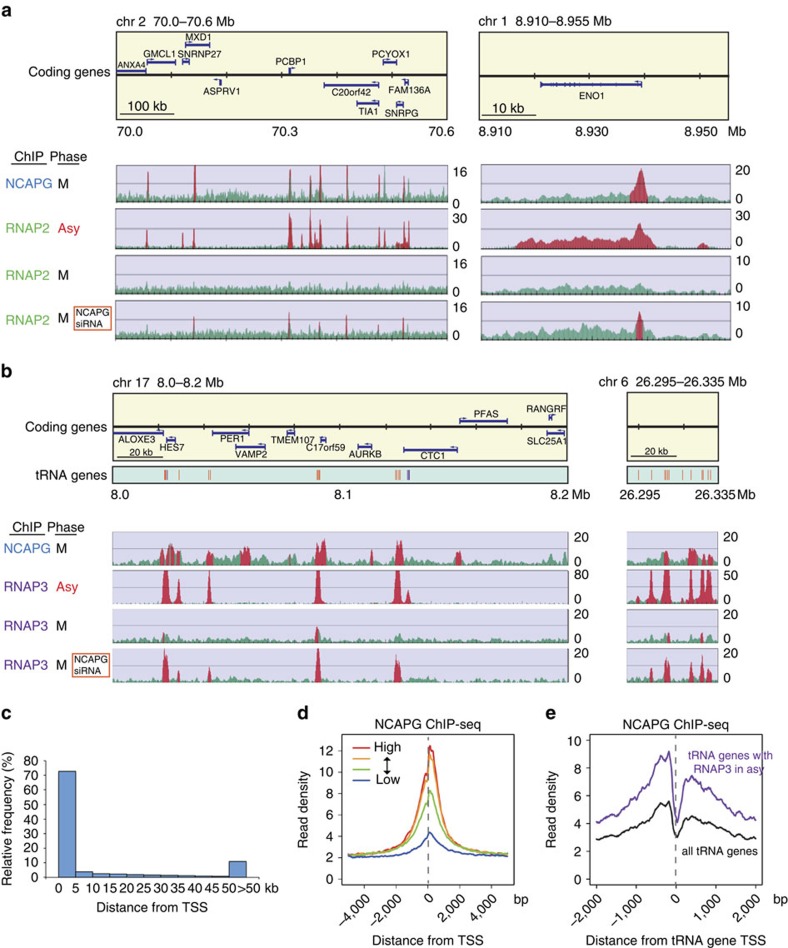
Binding of human condensin I complex to the TSS of active genes transcribed by RNAP2 and RNAP3. (**a**,**b**) ChIP-seq profiles of condensin I (using an antibody against NCAPG), RNAP2 (using 8WG16 monoclonal antibody, **a**) and RNAP3 (using monoclonal antibody against RPC32 subunit, **b**) in HeLa cells under the indicated conditions. M, cells arrested in prometaphase by nocodazole; Asy, asynchronous cells. Where indicated, the NCAPG subunit was depleted by siRNA treatment (Supplementary Fig. 8a). The upper boxes show the positions of protein-coding genes, and the lower boxes in **b** indicate the positions of tRNA genes (those within ORFs are shown in purple; others are shown in orange). The four sets of graphs in **a** and **b** show ChIP-seq profiles. The *y* axes of the ChIP-seq profiles indicate the normalized read intensity. Red indicates regions where enrichment determined by ChIP was statistically significant. (**c**) Distance of condensin I peaks from TSSs. More than 70% of NCAPG-binding sites are localized within ±5 kb of a TSS. (**d**) Level of condensin I binding during mitosis correlates with level of transcription during interphase. All RefSeq genes were ranked and divided into four equal groups based on their expression levels (high to low) in asynchronous cells, and averaged condensin I binding profiles around the TSS for each group were calculated. (**e**) Averaged mitotic NCAPG ChIP-seq profiles around tRNA genes. The profile for tRNA genes associated with RNAP3 in asynchronous cells is shown in purple.

Our findings in fission yeast prompted us to investigate the chromosomal localization of condensin in human cells. Most metazoan species possess two types of condensin complex, condensin I and II, with condensin I being more closely related to condensin in yeast^9^. Moreover, unlike condensin II and similar to fission yeast condensin, condensin I is associated with chromatin only during M phase^28^,^41^. We thus focused on condensin I. We first determined its genome-wide binding profile in prometaphase HeLa cells by ChIP-seq^22^,^23^ using an antibody against NCAPG (also known as CAP-G), a subunit specific to condensin I (ref. 42). The chromosome-wide distribution of NCAPG indicates that condensin I was enriched around the centromeres (Supplementary Fig. 7a), as reported in refs 15, 41. In addition, the condensin I profile showed a total of 7,825 distinct binding peaks along the chromosome arms (Fig. 6a). Close inspection revealed that most peaks overlapped with the transcription start sites (TSSs) of a subset of genes. More than 70% of detected peaks were located within ±5 kb of the TSS of an RNAP2-driven gene (Fig. 6c and Supplementary Fig. 7b). It is noteworthy that such TSS-proximal regions account for only 9.1% of the genome. The obtained condensin I binding profile was successfully validated by ChIP-qPCR. This binding was (i) more prominent at detected peaks than at sites distant from the peaks; (ii) more noticeable in mitotic cells than in cells at G1, which is consistent with the mitosis-specific association of condensin I with chromatin^41^; and (iii) diminished in NCAPG-depleted cells (Supplementary Fig. 8a–c), thereby verifying the specificity of the antibody used. Accumulation of condensin I at TSSs has also been reported recently in chicken DT40 cells^15^. In human cells, RNAP2 is enriched at the TSS of many genes because of the so-called RNAP2 promoter proximal pausing^43^. We found that condensin I peaks in prometaphase cells were associated with RNAP2 peaks in asynchronous cells, and the degree of condensin I binding at these peaks correlated nicely with the expression level of the corresponding genes in interphase cells (Fig. 6a,d). Therefore, as in fission yeast, human condensin I is localized at actively transcribed parts of the genome. Mitotic transcription is, however, repressed more strongly in human cells than in fission yeast^44^, as confirmed by RNAP2 ChIP-seq and RT–qPCR analysis in mitotic cells (Fig. 6a and Supplementary Fig. 8d). Moreover, transcription inhibitors caused no dissociation of condensin I from human mitotic chromosomes (Supplementary Fig. 8e). In addition, mitotic depletion of the TATA-binding protein (TBP), an essential component of the pre-initiation complex that is required for transcription initiation^32^, did not alter condensin I binding to TSSs (Supplementary Fig. 8f,g). Therefore, the binding of human condensin I at TSS regions is likely to be dependent on the chromatin structure or modifications specific to active genes, rather than transcription *per se* (see below).

In addition to RNAP2-dependent protein-coding genes, condensin I was detected around a subset of tRNA genes (164 sites, corresponding to 26% of human tRNA genes; Fig. 6b). As for the protein-coding genes, condensin I-bound tRNA genes were strongly expressed in asynchronous cells, but not in mitotic cells, as revealed by RNA polymerase III (RNAP3) ChIP-seq (Fig. 6b,e).

### Targeting of unwound DNA at TSSs by condensin I

**Figure 7:**
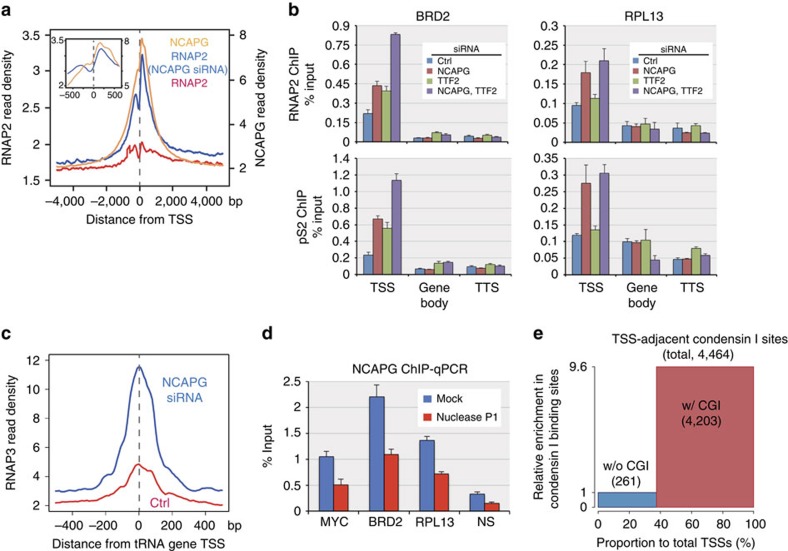
Recognition of ssDNA and expulsion of RNAPs from TSSs by human condensin I. (**a**) Appearance of RNAP2 ChIP-seq peaks at TSSs in condensin I−depleted cells. Mitotic ChIP-seq profiles of NCAPG and RNAP2 around TSSs (within ±5 kb) were averaged over all RefSeq genes and plotted. The *y* axis indicates the normalized read density. Profiles of NCAPG and RNAP2 ChIP-seq as well as RNAP2 ChIP-seq in NCAPG-depleted cells are shown. The inset provides a magnified view of the region ±500 bp from the TSS. (**b**) ChIP-qPCR of RNAP2 (detected by monoclonal antibody 8WG16) and active RNAP2 (phosphorylated at Ser2 in the C-terminal domain repeats, pS2). The NCAPG condensin I subunit and TTF2 were depleted by siRNA treatment, both individually and in combination. Binding to two gene loci (BRD2 and RPL13) was measured at the TSS, TTS and midpoint of the gene (gene body). NCAPG knockdown promoted binding of RNAP2 at the TSS, but not at the gene body or TTS, indicating that condensin I depletion is insufficient to de-repress mitotic gene expression. (**c**) Appearance of RNAP3 ChIP-seq peaks at the TSS of tRNA genes in condensin I-depleted cells. Averaged mitotic RNAP3 ChIP-seq profiles around tRNA genes in control and NCAPG-depleted cells are shown. (**d**) Treatment of condensin I-bound DNA fragments with nuclease P1. DNA fragments purified using NCAPG ChIP were treated with P1 on beads and then eluted and measured by qPCR. Condensin I-bound DNA was sensitive to P1 nuclease. NS represents a non-condensin-binding site (as a control). (**e**) Selective binding of condensin I to TSSs adjacent to CGIs. More than 90% of the condensin I binding sites adjacent to TSSs were located close to CGIs (within 1 kb, red). Such CGI-associated TSSs account for only ∼60% of TSSs in the genome, indicating an ∼9.6-fold enrichment of CGI-associated TSSs compared with non-CGI TSSs (blue) in condensin I−binding sites. Error bars in this figure represent s.d. (*n*=3, technical replicates in qPCR). Ctrl, control.

The association of condensin I with active genes led us to investigate whether it is involved in mitotic repression of transcription. RNAP2 ChIP-seq/ChIP-qPCR in mitotic cells revealed that NCAPG depletion resulted in the appearance of RNAP2 peaks at condensin-bound TSSs (Figs 6a and 7a, and Supplementary Fig. 9a,c,d). Cohesin (detected by RAD21 subunit ChIP^22^) was primarily in the chromosome-free, soluble fraction in normal mitotic cells and showed little or no increase in binding in NCAPG-depleted cells (Supplementary Fig. 9b), implying that the loading imposed by condensin depletion was specific to RNAP2. The increased binding of RNAP2 was unlikely to be an off-target effect of small interfering RNA (siRNA), because NCAPG knockdown by another siRNA oligo caused a similar enhanced RNAP2 binding (Supplementary Fig. 9c). Binding of RNAP2 was not seen in the middle region or TTS of the same genes (Fig. 7b), indicating that other mechanisms suppress the initiation-to-elongation switch of RNAP2 and/or promote dissociation of elongating RNAP2 in mitosis. Transcription termination factor 2 (TTF2) was at least partly responsible for these processes (Fig. 7b), as reported^45^. Consistently, RT–qPCR analysis confirmed that there was no marked increase in transcription of condensin I-bound genes as a result of NCAPG depletion (Supplementary Fig. 8d). In addition to RNAP2, RNAP3 was found to localize at condensin I-bound tRNA genes in NCAPG-depleted mitotic cells (Figs 6b and 7c, and Supplementary Fig. 9e). We therefore conclude that condensin I prevented recruitment of RNAP2 and RNAP3 to the promoters of active genes.

Finally, we examined whether human condensin I recognizes ssDNA. DNA purified by NCAPG ChIP was sensitive to nuclease P1, indicating that condensin I-bound DNA contains ssDNA regions (Fig. 7d). It was recently reported that promoters with CpG islands (CGIs) form long and stable R-loop structures immediately downstream from their TSSs^46^, which presumably expose the unwound ssDNA segment. We found that >90% of condensin I binding sites adjacent to TSSs are located within 1 kb of CGIs, representing a 9.6-fold enrichment of CGI-associated TSSs over TSSs without CGI in the condensin I binding sites (Fig. 7e). Similar preferential binding of condensin I to promoters with CGI was also observed in chicken DT40 cells^15^. The selective binding of condensin I to TSSs with CGIs is consistent with the possibility that condensin I recognizes ssDNA segments contained in these R-loop structures. Condensin I may function to accommodate this unusual structure with an unwound DNA segment in condensed chromatin.

## Discussion

In this study, we demonstrated transcription-dependent preferential binding of fission yeast condensin to actively transcribed genes on mitotic chromosomes. Transcription-dependent association of fission yeast condensin with active genes has also recently been reported^47^. It was claimed recently that highly transcribed genes can be associated with false-positive ChIP signals in budding yeast, as GFP (a control protein without DNA-binding capability) showed ChIP-seq peaks at active genes when localized in the nucleus^48^. In our study, however, the ChIP-seq profile of NLS-GFP-PK protein in mitotic fission yeast cells had a very poor correlation with the condensin profile, indicating that the condensin distribution profile was genuine. Furthermore, we found that human condensin I was bound to active genes at TSSs, and a similar distribution has been reported for condensin I in chicken DT40 cells^15^ and for condensin II in *C. elegans* embryos^16^. Thus, preferential association with actively transcribed regions seems to be a general feature of condensin complexes that is conserved in many eukaryotic species. Our analyses with nuclease P1 strongly suggest that both fission yeast condensin and human condensin I target unwound DNA segments produced by transcription at protein-coding genes. In contrast, a variety of distinct requirements have been described for condensin binding to centromere, rDNA and tRNA gene loci^19^,^25^,^49^,^50^,^51^,^52^. The general, locus-specific and species-specific mechanisms of condensin recruitment remain to be elucidated.

In budding yeast, mitotic transcription of specific loci (that is, rDNA and telomeric repeats) can have negative effects on the segregation of these loci^50^,^53^,^54^. Our results revealed that transcription of protein-coding genes by RNAP2, which occurs throughout the genome, was detrimental to faithful nuclear division when condensin function was compromised. We further demonstrated that the amount of chromosome-bound RPA in mitotic cells correlated positively with the degree of chromosome missegregation. Given that a core subcomplex of condensin promotes renaturation of ssDNA *in vitro* and that an RPA mutant with reduced affinity to ssDNA rescues a condensin mutant^35^,^39^, these results strongly suggest that what impedes chromosome segregation is the unwound DNA structure produced by transcription and stabilized by RPA. We propose that condensin recognizes and rewinds unwound DNA segments during assembly of condensed chromosomes.

**Figure 8:**
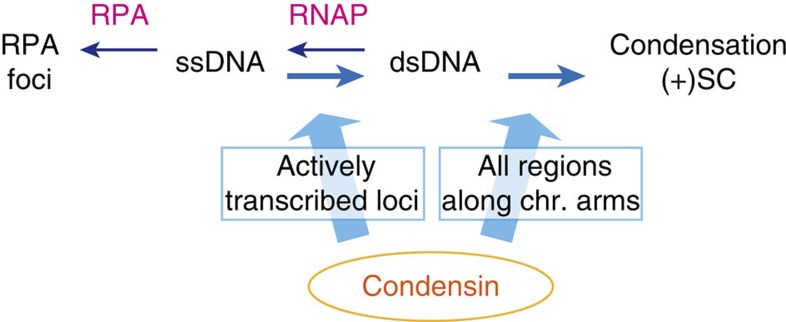
A model for physiological functions of condensin. At actively transcribed loci, condensin restores ssDNA produced during transcription to double-stranded DNA (dsDNA). This elimination of unwound DNA is a prerequisite for introducing global positive supercoiling ((+)SC) into DNA, which is presumably another physiological role of condensin and is executed at all regions along the chromosome (chr.) arms. (+)SC is expected to promote chromosome compaction and resolution, thereby contributing to assembly of condensed chromosomes^3^,^12^.

Restoration of unwound DNA by condensin can be readily incorporated into the above-mentioned model for the role of condensin in condensation, in which condensin reorganizes chromatin by introducing positive superhelical torsion into (or ‘overwinding') DNA. Before positive superhelical torsion can be globally introduced into DNA, unwound DNA structures must first be fixed. This would explain why unwound DNA caused by transcription^37^ interferes with condensation and why intense condensin activity is required at transcribed genes (Fig. 8). Our ChIP-seq/ChIP-qPCR analyses suggest that there are two types of condensin binding on chromosomes: predominant binding that is specific to active genes, and modest uniform binding along the chromosome arms (Supplementary Fig. 1d,f). The latter basal-level binding may reflect condensin activity to globally change DNA topology into an overwound configuration via positive supercoiling (Fig. 8).

The detected peaks of human condensin I were much sparser than those of fission yeast condensin (average peak-to-peak distance of 370 kb for human and 3.8 kb for fission yeast). Chicken condensin I peaks also show a similar sparse distribution^15^. It is possible that, because of mitotic repression of transcription in human cells, only ssDNA contained in stable structures such as R-loops at CGIs might persist in mitotic chromosomes and become the dominant binding sites of condensin I. This explanation could also give account of the differences of human condensin I binding properties from those of fission yeast condensin (that is, preference for TSSs over TTSs and insensitivity to transcriptional inhibitors). Similar to fission yeast condensin, human condensin I also had basal-level binding outside the enriched sites (Supplementary Fig. 8c). Condensin I might play an even more significant role at non-transcribed regions on human chromosomes. Although we found no evidence of transcription repression by fission yeast condensin, the rewinding of unwound DNA is a potential mechanism for regulating transcription. Indeed, human condensin I prevented the recruitment of RNAP2 and RNAP3 to promoters. Other isoforms of condensin complexes are also involved in large-scale transcriptional repression^55^,^56^. Recently mouse condensin II was shown to bind to active enhancers and promoters, and to be required for normal levels of gene expression in interphase embryonic stem cells^57^. Modulation of DNA configuration might underlie the mechanisms of transcriptional regulation by condensin complexes.

## Methods

### Yeast strains and culture

All yeast strains were *S. pombe* haploid wild-type 972 *h*^–^ or its derivatives and are listed in Supplementary Table 2. The media used for cell culture were complete yeast extract peptone dextrose (YPD) or minimal Pombe Minimal Glutamate (PMG)+supplements^58^. The endogenous *cut14*^+^ gene was epitope-tagged with nine copies of PK by plasmid integration^28^. An ∼700-base pair (bp) C-terminal segment of the gene fused with the PK epitopes was cloned into a pBluescript II KS(+) vector carrying *ura4*^+^ or *kanMX*. The resulting plasmid was linearized by digestion with EcoRI and integrated into the corresponding chromosomal locus by homologous recombination. PK-tagging of *rpb5*^+^ and GFP- and PK-tagging of *ssb1*^+^ were performed similarly. To create the Cnd2 shut-off strain (R81-^3FL^ Cnd2), the native promoter was replaced with a thiamine-repressible Rep81 promoter (an attenuated version of the *nmt1* promoter)^58^ by plasmid integration (Supplementary Fig. 4a). A Rep81 promoter followed by three FLAG epitopes and an ∼800-bp N-terminal fragment of Cnd2 was cloned into pBluescript II KS(+) carrying *kanMX*. The resulting plasmid was linearized at the *Bsp*1407I site within the Cnd2 fragment and then integrated into the endogenous *cnd2*^+^ locus by homologous recombination. Tagging and promoter replacement by plasmid integration were verified by colony PCR and immunoblotting. Deletion of *med6* was performed as described^59^ using a *kanMX* or *natMX* marker, and accurate disruption was confirmed by colony PCR.

For control ChIP-seq analysis using a non-DNA-binding protein, GFP fused with the SV40 large T-antigen nuclear localization signal (PKKKRKV) at the N terminus and with nine PK epitopes at the C terminus was placed under control of the *adh1* promoter and cloned into the pKY201 plasmid, which consists of pBluescript II SK(−) and the *aur1*^r^ marker (Takara Bio). The resulting plasmids were linearized at the EcoT22I site within the *aur1*^r^ marker and integrated into the endogenous *aur1*^+^ locus. Proper integration was verified by colony PCR.

Strains that possess an additional chromosomal copy of *cnd2* to express GFP-tagged wild-type Cnd2 or mutant Cnd2 3A (in which S5, S41 and S51 were replaced with alanines) have been described^25^. To visualize tubulin, a sequence encoding mCherry was fused to the N terminus of *atb2*^+^, cloned under control of the *adh15* promoter (a weaker version of the *adh1* promoter) and integrated into the C locus using the *hphMX* marker^25^.

For the analysis of mitotic promoter-driven condensin localization with the GFP reporter gene, *slp1* and *meu13* promoters (−1,657 to −1 and −926 to −1 relative to the coding sequence, respectively) were fused with the GFP ORF and *adh1*^+^ 3′ untranslated region (UTR) sequences and were cloned into pKY201. A mutated *slp1* promoter that harbours a 7-nucleotide deletion (−1,019 to −1,013, designated as TATA-less *slp1* promoter) was also cloned. The resulting plasmids were linearized and integrated into the endogenous *aur1*^+^ locus as described above. Proper integration was verified by colony PCR.

To arrest cells at prometaphase, *nda3* cold-sensitive mutant cells^17^ were cultured in YPD at 33 °C to a density of 5−6 × 10^6^ cells per ml and then were shifted to a restrictive temperature of 20 °C for 6 h. Cell cycle arrest was assessed by 4,6-diamidino-2-phenylindole (DAPI) staining of glutaraldehyde-fixed cells; proper arrest was indicated by a decreased frequency of cells with two evenly segregated nuclei (typically <5%) and the appearance of cells with a condensed nucleus (>80%). To obtain prometaphase-arrested Cnd2-depleted cells, *nda3* R81-Cnd2 cells were cultured in PMG+supplements (a thiamine-free medium) at 33 °C to a density of 3 × 10^6^ cells per ml. They were then transferred to thiamine-containing YPD medium at 33 °C for 3 h to repress Cnd2 expression and were shifted to 20 °C for an additional 6 h. To monitor Ssb1-GFP focus formation in mitotic cells (Fig. 5c,d), the cells were moderately synchronized by hydroxyurea (HU) block and release to increase the proportion of mitotic cells. Cells were first arrested in early S phase by incubating in YPD with 11 mM HU at 26 °C for 3.5 h, released into HU-free YPD and incubated at 26 °C for 30 min and finally transferred to 34 °C for an additional 1 h to let the majority of cells enter mitosis at the restrictive temperature of the *cut14* mutant. Ssb1 foci were counted in early mitotic cells with short spindles (visualized by mCherry-tagged α-tubulin).

### Human cell culture and synchronization

HeLa cell were cultured in DMEM supplemented with penicillin–streptomycin–L-glutamine solution (Wako), 10% fetal bovine serum and 20 mM HEPES-KOH (pH 7.4). To obtain cells arrested at prometaphase, HeLa cells were first synchronized using a double thymidine block (14–16 h in the presence of 2 mM thymidine, an 8-h release and then 16 h in the presence of 2 mM thymidine) and then were released into thymidine-free medium for 7 h and arrested at prometaphase by treatment with nocodazole (50 ng ml^-1^, Calbiochem) for 3 h. Mitotic cells were collected by shake-off. For RNAP2 ChIP-seq analysis, cells after the second thymidine block and release were arrested at late G2 phase by treatment with Cdk1 inhibitor RO3306 (8 μM, Calbiochem) for 2 h and then were released and re-arrested at prometaphase by treatment with nocodazole for 2 h, before collection of mitotic cells by shake-off; this excluded non-mitotic cells from the analysis as much as possible. To obtain G1 cells, HeLa cells arrested at prometaphase by nocodazole treatment were released synchronously from arrest for 2 h. The synchronization was assessed by flow cytometric analysis. The used HeLa cell line was provided by Dr. T. Hirota (The Cancer Institute of Japanese foundation for cancer research [JFCR]). Cells stably expressing GFP-tagged CENPA have been described^60^.

### RNA interference

NCAPG, TBP and TTF2 siRNA transfections were performed using Lipofectamine RNAiMAX (Life Technologies) in accordance with the manufacturer's protocol, using a final RNA duplex concentration of 50 nM. To deplete these proteins in synchronized cell culture, transfections were carried out 8 h before the first thymidine treatment. The siRNA sense sequences used for targeting NCAPG were 5′-AAUAAGACGAGAAAGAAUCCUGCUG-3′ and 5′-UAAAUAGUCUGCAUAUACUACAGGC-3' (no. 2 oligo in Supplementary Fig. 9c). The siRNA sense sequences used for targeting TBP and TTF2 (ref. 45) were 5′-GGGAGCUGUGAUGUGAAGUUUCCUA-3′ and 5′-GGAAAGAGCUUCUACGUGU-3′, respectively.

### Antibodies and reagents

Monoclonal anti-PK (SV5-Pk1 clone, AbD Serotec), monoclonal anti-FLAG (M2 clone, Sigma), polyclonal anti-GFP (Living Colors Full-Length A.v. Polyclonal Antibody, Clontech for yeast; TP401, Torrey Pines Biolabs for HeLa cells), monoclonal anti-α-tubulin (B-5-1-2 clone, Sigma), polyclonal anti-histone H3 (ab1791, Abcam), monoclonal anti-TBP (MAb-TBPCSH-100, diagenode) and monoclonal anti-Pol III RPC32 (sc-21754, Santa Cruz Biotechnology) were used for immunoblotting, immunostaining and ChIP. For RNAP2 immunoblotting and ChIP, monoclonal anti-RNAP2 C-terminal domain repeat (8WG16 clone, Abcam) was used. This antibody can recognize both unphosphorylated and phosphorylated forms of RNAP2 (Supplementary Fig. 5b), although presumably it has higher affinity for unphosphorylated one. Monoclonal antibodies against RNAP2 phosphorylated at Ser2 or Ser5 (anti-pS2 and anti-pS5, respectively) were prepared and provided by Dr. H. Kimura (Tokyo Institute of Technology)^61^. The specificity of these antibodies for activated fission yeast RNAP2 was assessed using cells treated with the transcription inhibitor 1,10-phenanthroline (Supplementary Figs 2b and 5b). Polyclonal antibodies against human NCAPG and RAD21 have been described^22^,^42^. For mock immunoprecipitation in human ChIP, normal (nonspecific) rabbit immunoglobulin G (IgG; no. 2797, Cell Signaling) was used. Anti-mouse IgG conjugated with Alexa Fluor 488 (Life Technologies) was used as secondary antibody in immunofluorescence. Antibody dilutions in immunoblotting were 1:2,000 (anti-FLAG), 1:1,000 (anti-α-tubulin), 1:1,000 (anti-histone H3), 1:2,000 (anti-TBP), 1:2,000 (anti-RNAP2), 1:6,000 (anti-pS2), 1:6,000 (anti-pS5) and 1:1,000 (NCAPG). Antibody dilution in cell immunostaining was 1:500 (anti-pS2). Thiolutin and 1,10-phenanthroline (Sigma) were used as transcription inhibitors in fission yeast. In liquid culture, 1,10-phenanthroline was added at 120 μg ml^−1^ 30 min before cell fixation, unless otherwise specified. Triptolide (Enzo Life Sciences) and SNS-032 (Selleck Chemicals) were used as RNAP2-specific transcription inhibitors in HeLa cells^62^,^63^.

### Chromatin immunoprecipitation

ChIP of fission yeast cells was performed as described^21^ with several modifications. ∼5 × 10^8^ yeast cells were fixed with 1% formaldehyde for 25 min at room temperature, followed by an additional 5 min with glycine added at a final concentration of 250 mM. Fixed cells were washed and then lysed in 1.6 ml of lysis buffer^21^ containing 0.5-mm diameter zirconia beads (YZB05, Yasui Kikai) for 10 min at 0 °C with rigorous shaking (2,700 r.p.m. on a Multi-Beads Shocker, Yasui Kikai). Fixed chromatin in the resultant lysate was sonicated using Branson Sonifier 250D to generate DNA fragments with a mean length of 500–700 bp, clarified by centrifugation (13,000*g*, 5 min, two times) and incubated with antibody beads (Dynabeads Protein A (Life Technologies) complexed with ∼20 μg antibody) for 14 h at 4 °C. The beads were then washed rigorously, and material captured on the beads was eluted by Tris-EDTA (TE) buffer containing 1% SDS. The recovered material was incubated at 65 °C overnight to reverse crosslinks, treated with RNase A and proteinase K, and purified by Qiagen PCR purification kit (Qiagen). ChIP of HeLa cells was performed as described^22^. In brief, ∼1 × 10^7^ cells were crosslinked with 1% formaldehyde for 10 min at room temperature, followed by an additional 5 min with glycine added at a final concentration of 125 mM. Fixed cells were lysed and treated with 1,000 gel units Micrococcal nuclease for 15 min at 37 °C, followed by sonication for chromatin shearing. Lysate containing fragmented chromatin was incubated with antibody beads (Dynabeads Protein A or Dynabeads anti-Mouse IgG (Life Technologies) complexed with antibody) for 14 h at 4 °C. The beads after immunoprecipitation were processed as in the case of yeast ChIP. We previously found that dual-crosslinking using ethylene glycol bis(succinimidyl succinate) and formaldehyde^64^ increased the signal-to-noise ratio of the ChIP-seq profile of NCAPG on human mitotic chromosomes without affecting the distribution pattern. Hence, for ChIP-seq analysis of human NCAPG, HeLa cells were crosslinked with 1.5 mM ethylene glycol bis(succinimidyl succinate) for 45 min at room temperature, followed by 1% formaldehyde for 20 min, and then were quenched with 200 mM glycine for 10 min.

### Quantitative PCR

Analysis of ChIP-purified DNA by qPCR was carried out using real-time PCR systems 7500 and StepOnePlus (Life Technologies) and KAPA SYBR Fast qPCR kit (KAPA Biosystems). The used primer sets are listed in Supplementary Table 3.

### ChIP-seq analysis

High-throughput sequencing was carried out using SOLiD 3, SOLiD 4, SOLiD 5500 (Applied Biosystems) and HiSeq 2000 (Illumina) systems. DNA before and after ChIP (input and ChIP DNA, respectively) was processed and sequenced per the manufacturer's instructions. Briefly, DNA was sheared to an average size of ∼150 bp by ultrasonication (Covaris), end-repaired, ligated to sequencing adapters, amplified, size-selected and sequenced to generate single-end 50-bp reads. Reads were aligned to the *S. pombe*^65^ or human (UCSC hg19) genome sequence using Bowtie^66^. Information and statistics regarding sequencing and mapping are summarized in Supplementary Table 4. Mapping results were processed and converted to genome-wide protein distribution profiles (ChIP-seq profiles) using parse2wig and DROMPA software as described^23^.

For *S. pombe* ChIP-seq analysis, multiply aligned reads were processed without PCR bias filtering to allow analysis within repetitive DNA elements, including centromeres and rDNA loci. DROMPA produces a list of relative enrichment ratios (rERs), each of which is a ratio of the number of ChIP sequence reads mapped at a specific 10-bp genome segment to the number of input sequence reads mapped at the same genome site, smoothened with a 100-bp size window and normalized to make the genome-wide average of rER values equal to 1. The rER value accurately reflects the degree of enrichment in the ChIP procedure (as indicated in Fig. 1b). The rER values were plotted along chromosomes using an Integrative Genomics Viewer^67^. Regions poorly covered by the input sequencing (that is, <20% of the average input read depth) were omitted from analysis (these typically corresponded to <3% of the genome), because they can potentially show pseudo-positive signals in the rER profile. Peak calling by DROMPA was carried out using default parameters^23^, but with rER values of >2.0 (options; -ethre 2 -ithre 2) for moderately enriched sites or >5.0 (-ethre 5 -ithre 5) for strongly enriched sites. Gene annotations and UTR information for fission yeast were obtained from PomBase^68^ and used to deduce TSSs and TTSs. For genes with no UTR information, the mean 5′- and 3′ UTR lengths were used (293 and 430 bp, respectively)^68^ for this purpose. Heat maps, correlation plots and averaged (metagene) profiles of fission yeast ChIP-seq results were calculated from rER data sets and visualized with homemade scripts in Perl and R. For correlation analysis between ChIP-seq and ChIP-qPCR results (Fig. 1b), the minimum rER value within each qPCR amplicon was used as the representative rER value.

For human ChIP-seq analyses, uniquely mapped reads were processed by parse2wig and DROMPA. Normalized read intensities of ChIP samples, instead of rER values, were used to visualize human ChIP-seq profiles as reported^23^, because the relatively low read depth available for human input samples causes large stochastic fluctuations in the rER profile. In peak calling, both ChIP and input sequence results were used to eliminate pseudo-binding signals in input samples, as described^23^. Peak calling of human RNAP2 ChIP-seq by DROMPA was carried out using default settings. For NCAPG ChIP-seq peak calling, a stricter threshold, *R*_*x*_ >7.0 (option; -ipm 7), was used to keep the false discovery rate of detected peaks below 5%, where *R*_*x*_ is the normalized read intensity in the modified reads per kilobase per million mapped reads (RPKM)^23^. RNA-seq data of asynchronous HeLa cells (GMS765402 in Gene Expression Omnibus) were used to classify genes based on their expression level (Fig. 6d). Annotations for CGIs were obtained from the UCSC Genome Bioinformatics site (http://hgdownload.soe.ucsc.edu/goldenPath/hg19/database/).

### Sample–sample normalization of ChIP-seq data

For quantitative comparison of ChIP-seq data sets between different strains or cultivation conditions (that is, Fig. 3e and Supplementary Fig. 2c), rER values must be normalized based on external standards, because the amount of DNA recovered by ChIP is expected to be different between samples, making the total read normalization implemented in DROMPA inadequate. We used ChIP-qPCR results measured at six or more sites as the external standards. In the case of Supplementary Fig. 2c, the ChIP-qPCR % input values obtained showed excellent correlation with ChIP-seq rER values at the corresponding sites in both samples (correlation coefficients >0.99). We re-scaled (multiplied by a constant) the rER data set for 1,10-phenanthroline–treated cells so that the regression line of the ChIP-qPCR versus ChIP-seq plot for treated cells corresponds to that for untreated cells. The excellent correlation between ChIP-seq and ChIP-qPCR measurements (*r*≥0.95 in all cases) assures the accuracy of this adjustment.

### Nuclease treatment of ChIP-purified DNA

DNA associated with Cut14-PK, histone H3 or Rad21-GFP was purified using the ChIP procedure as described. Before elution from antibody-bound magnetic beads, DNA on beads was treated with nuclease under the following conditions. All of the reactions were performed in a volume of 100 μl using input DNA derived from 1.5 × 10^8^ yeast cells. For nuclease P1 (Wako Pure Chemical Industries) treatment, 10 U enzyme was used in a buffer comprising 40 mM MES-Na, 400 mM NaCl, 0.2 × cOmplete-EDTA protease inhibitor cocktail (Roche), 50 μg ml^−1^ BSA and 1 μg ml^−1^ fragmented λ-DNA at pH 6.0 and incubated for 20 min at 42 °C. For RNase A (Roche) treatment, 50 μg enzyme was used in a buffer containing 10 mM Tris-Cl, 1 mM EDTA and 0.2 × cOmplete-EDTA protease inhibitor cocktail at pH 8.0, and incubated for 20 min at 37 °C. For RNase H (Takara Bio) treatment, 60 U enzyme was used in a buffer containing 40 mM Tris-Cl, 4 mM MgCl_2_, 0.2 × cOmplete-EDTA protease inhibitor cocktail and 50 μg ml^−1^ BSA at pH 8.0 and incubated for 20 min at 37 °C. On completion of the reactions, beads were collected and washed once with TE buffer and then DNA was eluted, reverse-crosslinked and purified as described^21^. For analysis of DNA binding to human NCAPG, DNA purified from 5 × 10^6^ cells was processed as described above.

### RNA isolation and RT–qPCR

Total RNA was isolated from fission yeast cells by phenol/chloroform/SDS extraction method^69^, then purified using RNeasy columns (Qiagen). To eliminate genomic DNA completely, on-column DNase digestion of RNA was carried out following the manufacturer's instructions. From HeLa cells, total RNA was isolated using Trizol (Invitrogen) and Nucleospin RNA kit (Macherey–Nagel) following the manufacturer's instructions. The isolated total RNA was converted to complementary DNA (cDNA) using the SuperScript III First-Strand Synthesis System for RT–qPCR (Life Technologies) using random primers (for yeast RNA) or using ReverTra Ace qPCR RT Master Mix (Toyobo; for HeLa cell RNA). cDNA was analysed by qPCR as described in the ‘Quantitative PCR' section. In Supplementary Fig. 2a, human GAPDH mRNA (Nippon Gene) was used as a spike-in control to normalize RNA extraction and reverse transcription efficiency. The β-actin gene was used as an internal control in human RT–qPCR analysis. The primer sets used are listed in Supplementary Table 3. The primers used to detect nascent human mRNAs have been described^70^.

### RNA-seq analysis

Poly(A)-containing mRNA molecules were isolated from total RNA, converted to cDNA with random primers using TruSeq RNA Sample Preparation kit v2 (Illumina) and sequenced on a HiSeq 2000 platform to produce 36-mer single-end reads. Reads were mapped, analysed and visualized as described in the ChIP-seq analysis section.

### Isolation and identification of suppressor mutations

A single colony of the *h*^*–*^ *leu1 cut3-477* strain was inoculated into 10 ml YPD medium and cultivated at 26 °C to a density of ∼1 × 10^7^ cells per ml. Cells were harvested, spread on a YPD plate and incubated at 34 °C (a restrictive temperature of the mutant) for 3 days. Although most cells ceased to divide, several formed colonies, presumably because of spontaneous suppressor mutations. One colony was collected as a phenotypic revertant of *cut3-477*. By repeating this process using independent initial colonies, a collection of *cut3* revertants was created. Genome DNA of each clone was sequenced by HiSeq 2000 to produce >60 million single-end, 50-bp-long reads. By comparing these sequences with the reference genome sequence as described^30^, the responsible suppressor mutation in each revertant was identified.

### Protein analysis

To prepare yeast cell extract for immunoblotting analysis, 5 × 10^7^ fission yeast cells were lysed in 50 μl ChIP lysis buffer^21^ supplemented with 1 × PhosSTOP phosphatase inhibitor cocktail (Roche) using the Multi-Beads Shocker at 4 °C. The resulting lysate was centrifuged at 2,000*g* for 1 min, and the supernatant was recovered, mixed with SDS–polyacrylamide gel electrophoresis sample buffer and boiled for 3 min. To obtain HeLa cell total extract, HeLa cells were lysed with lysis buffer containing 0.2% NP-40 as described^22^. To perform chromatin fractionation, HeLa cells were lysed in buffer containing 20 mM HEPES-KOH, pH 7.5, 100 mM NaCl, 10 mM KCl, 10% glycerol, 340 mM sucrose, 1.5 mM MgCl_2_, 0.25% Triton X-100, 1 mM dithiothreitol, 1 × cOmplete proteinase inhibitor cocktail (Roche) and 1 × PhosSTOP, and were kept on ice for 10 min. After the lysate was centrifuged at 1,500*g* for 5 min at 4 °C, the supernatant was recovered as the chromatin-free soluble fraction, and the pellet was resuspended in SDS–polyacrylamide gel electrophoresis sample buffer and boiled to give the chromatin fraction. Uncropped scans of protein immunoblotting data (Supplementary Figs 4b, 8a and 8g) are shown in Supplementary Figs 10 and 11.

### Cytology and immunostaining

To observe nuclear morphology, fission yeast cells were fixed with glutaraldehyde and stained with DAPI^29^. Immunofluorescence staining of yeast cells was performed using methanol fixation method^29^. To observe GFP and mCherry fluorescence signals, cells were fixed with cold methanol at −80 °C, rehydrated with PEM buffer^29^ and stained with DAPI. An Olympus BX-51 fluorescence microscope with DP-30 digital camera was used to observe fixed cells.

### Repeatability of experiments

All of the ChIP-seq profiles were verified by independent ChIP-seq experiments (for fission yeast Cut14-PK and human condensin I) and by independent ChIP-qPCR analyses measured at multiple genomic loci. All of the experiments utilizing qPCR, yeast genetics/cytology and immunoblotting were repeated independently at least twice, unless stated otherwise in each figure legend, with similar results. The averaged data of all the performed experiments (for Ssb1 focus formation assay) or one representative experiment is shown.

### Statistical analysis

Quantitative data are presented as mean±s.d. (for qPCR measurements) or mean±s.e.m. (for Ssb1 focus formation assay). Welch's *t*-test was used to compare two groups for independent samples, unless stated otherwise. Investigators were not blinded to allocation during experiments and outcome assessment.

## Additional information

**Accession codes:** ChIP-seq, RNA-seq and whole-genome sequencing data from this study are available from the Sequence Read Archive database under the accession numbers, SRP045410, SRP045412, SRP045414 and SRP045415.

**How to cite this article:** Sutani, T. *et al*. Condensin targets and reduces unwound DNA structures associated with transcription in mitotic chromosome condensation. *Nat. Commun.* 6:7815 doi: 10.1038/ncomms88815 (2015).

## Supplementary Material

Supplementary Information Supplementary Figures 1-11, Supplementary Tables 1-4 and Supplementary References
