# Supplementary material for: Condensin targets and reduces unwound DNA structures associated with transcription in mitotic chromosome condensation
Source: Nat Commun. 2015 Jul 23;6:7815. doi: 10.1038/ncomms8815 (PMC4525155; doi:10.1038/ncomms8815)
Supplement: Supplementary Information — Supplementary Figures 1-11, Supplementary Tables 1-4 and Supplementary References [file ncomms8815-s1.pdf]

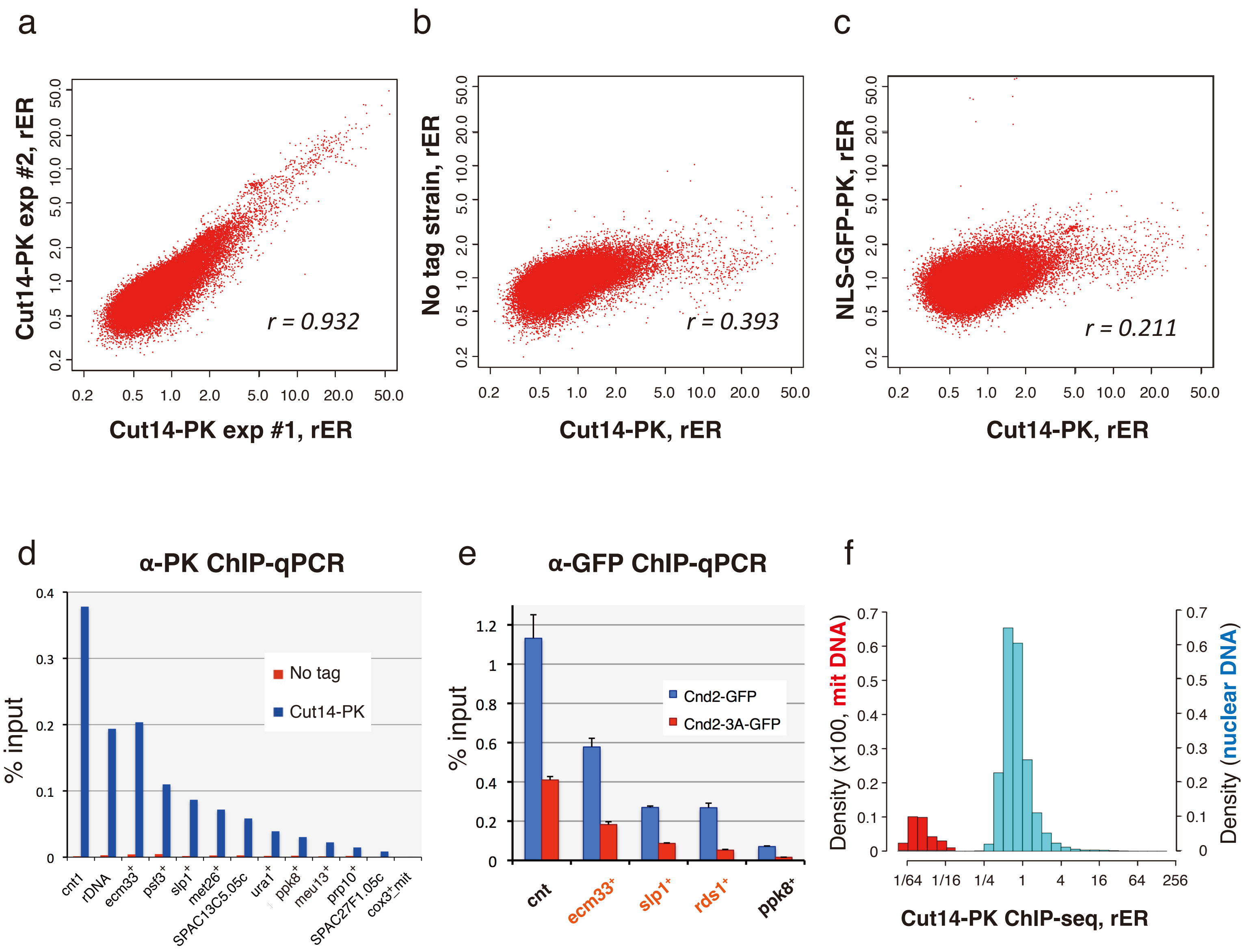

Supplementary Figure 1

Supplementary data for Figure 1 (identification of condensin binding sites in fission yeast mitotic chromosomes). (**a**) Correlation plot between Cut14-PK ChIP-seq results from two independent experiments. A good correlation was evident, indicating that the profile is experimentally reproducible. *r*, Pearson's correlation coefficient. (**b,c**) Correlation plots between Cut14-PK and control ChIP-seq experiments. In (b), an untagged strain was subjected to anti-PK ChIP. In (c), cells expressing a nuclear-localised, non-DNA-binding control protein with PK epitopes (NLS-GFP-PK) were subjected to anti-PK ChIP. Both showed poor correlation with Cut14-PK, precluding the possibility that the peaks observed in the Cut14-PK ChIP-seq were derived from Cut14-independent enrichment of DNA during anti-PK ChIP. (**d**) Verification of Cut14-PK ChIP-seq results by qPCR. DNA isolated by anti-PK ChIP from untagged (red) or Cut14-PK (blue) cells was measured by qPCR at 13 selected sites in the genome. Results are shown as ChIP efficiency values (% input). Each qPCR site is named after the nearby gene or genomic feature. cox3$^+$_mit is the cox3$^+$ gene on the mitochondrial genome. Sites are sorted from left to right according to the Cut14-PK ChIP-seq rER value at the corresponding position, in descending order. (**e**) ChIP-qPCR analysis of another condensin subunit, Cnd2. Wild-type and mutant Cnd2 subunits with a GFP tag (Cnd2-GFP or Cnd2-3A-GFP, respectively) were ectopically expressed in prometaphase cells, and chromosomal binding of condensin complexes with the expressed GFP-tagged subunits was measured by ChIP-qPCR. Wild-type Cnd2 showed preferential binding at the identified condensin binding sites (orange). Cnd2-3A mutant protein lacks target sites for mitosis-specific phosphorylation, has reduced affinity for chromatin, and is defective in promoting chromosome condensation[1,2]. Here, the Cnd2-3A mutant showed reduced binding at all sites tested, implying that the observed binding reflects a physiological role of condensin. Error bars represent s.d. (n = 2, technical replicates in qPCR). This experiment was performed once. (**f**) Distribution of Cut14-PK ChIP-seq rER values. Values corresponding to the nuclear genome (blue) and mitochondrial DNA (red) are shown separately. rER values for the nuclear genome are considerably higher than those for mitochondrial DNA, which presumably reflects nonspecific, background-level precipitation by anti-PK beads.

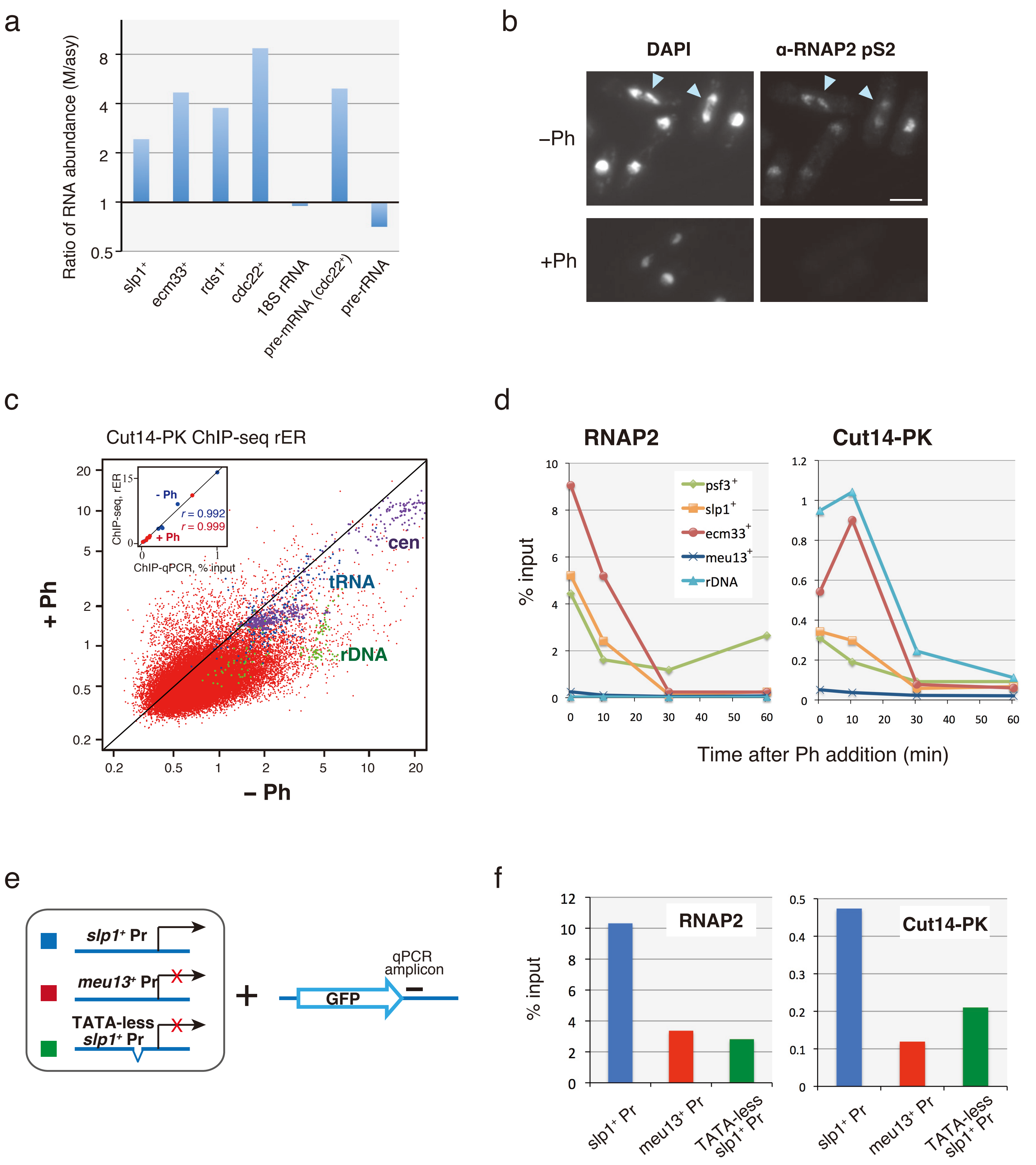

Supplementary Figure 2

Supplementary data for Figure 2 (transcription-dependent condensin binding at RNAP2-driven active genes). (**a**) Active transcription in fission yeast mitotic cells. Total RNA from asynchronous (asy) or prometaphase-arrested (M) cells was reverse-transcribed with random primers, and levels of the indicated mRNAs and rRNAs were measured by qPCR. mRNAs of the *slp1*$^{+}$, *ecm33*$^{+}$, *rds1*$^{+}$ and *cdc22*$^{+}$ genes, all of which are condensin binding sites, were more abundant in M-phase cells. Pre-spliced nascent transcripts (pre-mRNA) of *cdc22*$^{+}$ were also increased during M phase, indicating that a subset of genes is actively transcribed in fission yeast mitotic cells. Pre-rRNA is a nascent, pre-processed form of rRNA transcripts. (**b**) Association of active RNAP2 with chromatin in mitotic cells. Asynchronous fission yeast cells were immunostained with antibodies that specifically recognise RNAP2 phosphorylated at Ser2 in the CTD repeats (α-RNAP2 pS2). The signal colocalised with chromatin (revealed by DAPI staining), even in mitotic cells with segregating nuclei (arrowheads), indicating that active transcription continues in mitotic cells. Treatment with the transcription inhibitor, 1,10-phenanthroline (Ph) completely suppressed the antibody signal, verifying the specificity of the antibody. Scale bar, 5 µm. (**c**) Genome-wide correlation plot of Cut14-PK ChIP-seq data in untreated and Ph-treated prometaphase cells. Sample-sample normalisation of ChIP-seq data was performed using independent ChIP-qPCR measurement at six genomic loci (inset). Purple, green and blue dots correspond to centromere, rDNA and tRNA gene loci, respectively. Ph treatment reduced condensin binding at most condensin binding sites, without producing novel condensin-enriched regions. (**d**) Time course of Cut14-PK and RNAP2 binding to chromosomes in transcription-inhibited cells. Prometaphase-arrested cells were treated with Ph for 0, 10, 30 or 60 min, and the association of RNAP2 and Cut14-PK with chromatin was monitored by ChIP-qPCR. RNAP2 dissociation was observed within 10 min of Ph treatment, whereas Cut14-PK dissociation was observed at 30 min. (**e,f**) A mitotically active promoter is sufficient to recruit condensin on chromosomes. (e) Experimental design. A GFP reporter gene placed under the control of various promoters was integrated into a chromosome at the *aur1*$^{+}$ locus. The promoters tested were the mitotically active s*slp1*$^{+}$ promoter (Pr), the mitotically inactive *meu13*$^{+}$ promoter and a mutated *slp1*$^{+}$ promoter that lacks a TATA element and is presumably transcriptionally inactive. (f) qPCR results. RNAP2 ChIP-qPCR confirmed that only the native slp1+ promoter induced transcription, as expected. Cut14-PK ChIP-qPCR revealed the association of condensin only at the *slp1*$^{+}$ promoter−fused GFP gene. All the experiments in this figure were performed once.

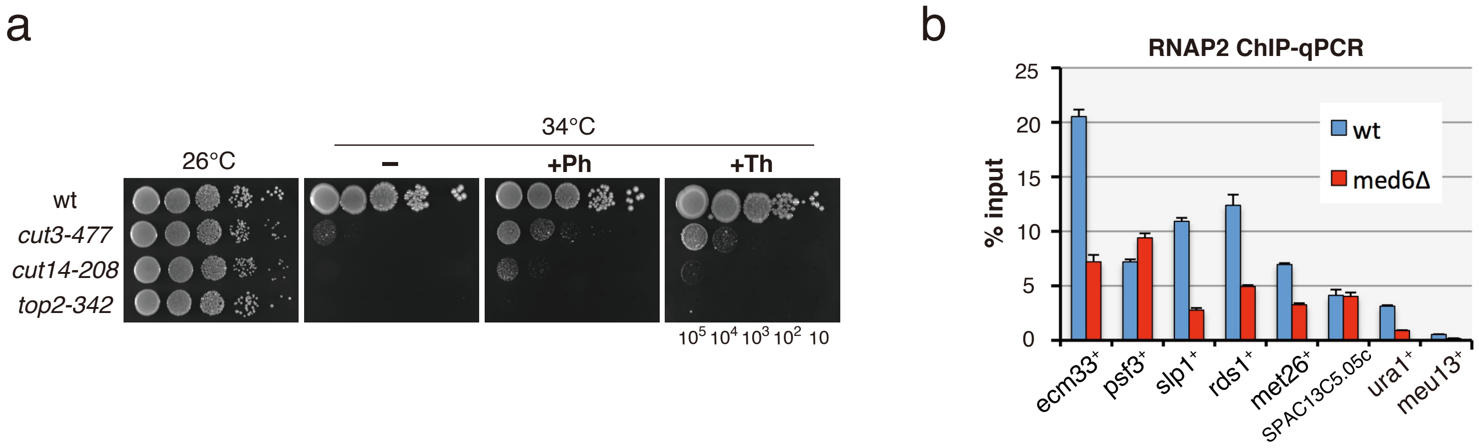

Supplementary Figure 3

Supplementary data for Figure 3 (suppression of the chromosome segregation defect in condensin mutants by transcription attenuation). (**a**) Serial dilution growth assay on YPD plates in the absence or presence of transcription inhibitors (Ph, 20 µg/ ml^{-1}; Th, 3 µg/ ml^{-1}) at the indicated temperatures. *cut3-477* cells have a mutation in the condensin subunit Cut3 (ref. 3). *top2-342* is a mutant of DNA topoisomerase II[2], another key player in chromosome condensation. Growth defects observed in condensin mutants at the restrictive temperature were partly rescued by these inhibitors. (**b**) ChIP-qPCR of RNAP2 in prometaphase-arrested wild-type and *med6*Δ cells. The *med6* deletion reduced chromatin binding of RNAP2 at five of seven active genes examined. Error bars represent s.d. (n = 2, technical replicates in qPCR).

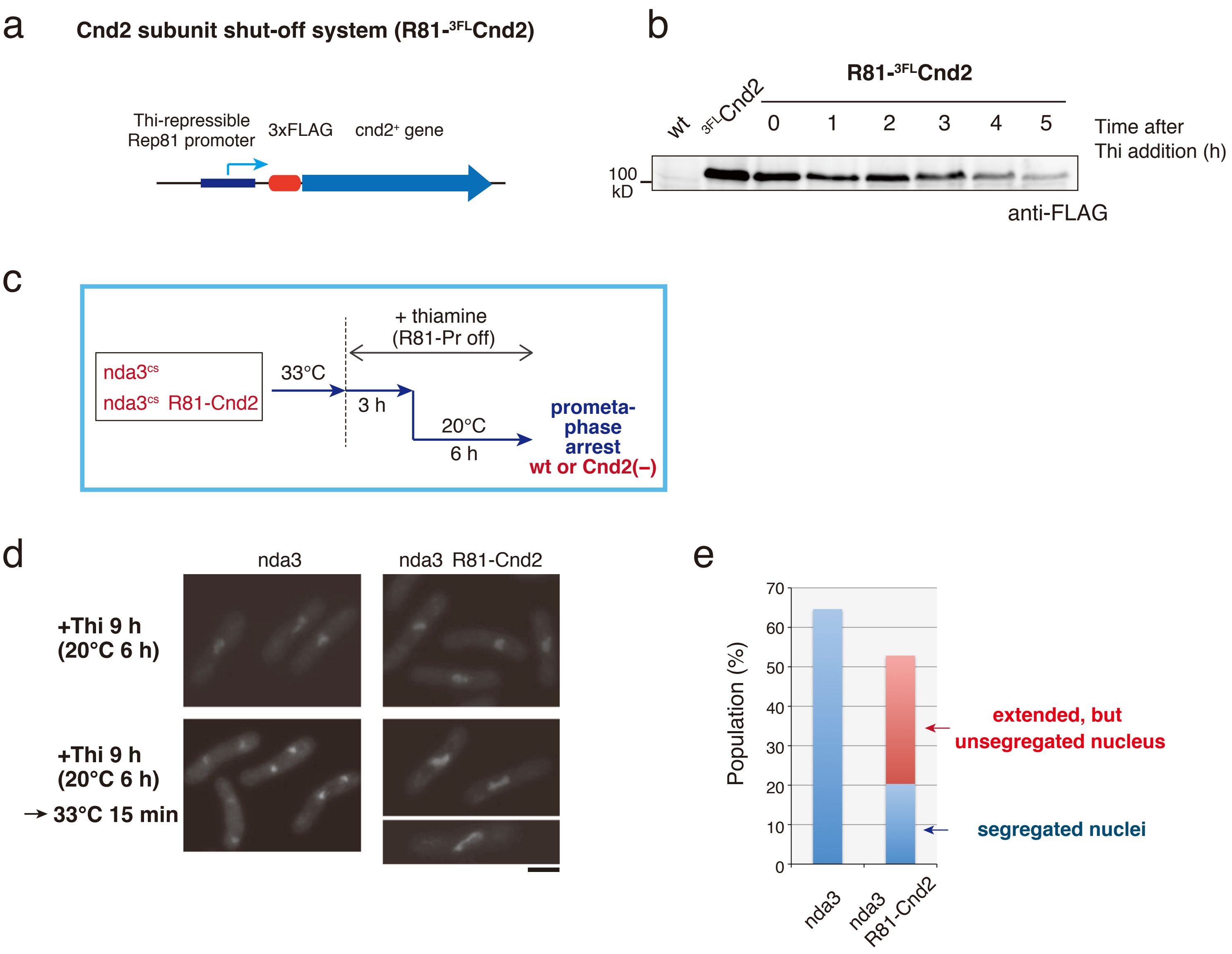

Supplementary Figure 4

Construction of the Cnd2 shut-off strain. (**a**) To conditionally repress expression of Cnd2, the native promoter was replaced with a thiamine (Thi)-repressible Rep81 promoter, an attenuated derivative of the *nmt1*+ promoter[4]. A 3×FLAG epitope was also integrated at the N terminus for protein detection. The resulting strain was designated as R81-3FLCnd2. (**b**) Cnd2 depletion revealed by immunoblotting. In the absence of thiamine (time 0), the level of Cnd2 in the R81-3FLCnd2 strain is comparable to that in 3FLCnd2, in which FLAG-tagged Cnd2 was placed under its native promoter. At 5 h after addition of thiamine to growing cultures of R81-3FLCnd2 cells (at 33° C), the amount of Cnd2 protein was reduced to ~10% of the initial amount. (**c**) Experimental scheme to obtain Cnd2-depleted, prometaphase-arrested cells. R81-3FLCnd2 cells harbouring a cold-sensitive (cs) *nda3* mutation were cultured in the presence of thiamine for 3 h at 33°C and then grown for an additional 6 h at 20°C to arrest cells at prometaphase. (d,e) Verification of Cnd2 depletion in prometaphase-arrested cells. (**d**) Prometaphase-arrested, Cnd2-depleted cells were prepared as described, fixed and analysed using DAPI staining. R81-3FLCnd2 cells showed less nuclear compaction than wild type, indicating defective condensation. When aliquots of cells were cultured for 15 min at 33 ° C to be released from the arrest before fixation, R81-3FLCnd2 cells showed extended but unsegregated nuclei like those seen in condensin mutants[3,5], whereas wild-type cells exhibited proper chromosome segregation. Taken together, these data indicate that condensin was reduced to a level that was insufficient to accomplish proper chromosome condensation. Scale bar, 5 µm. (**e**) Frequency of properly segregated and unsegregated nuclei in post-anaphase cells (15-min release from the arrest).

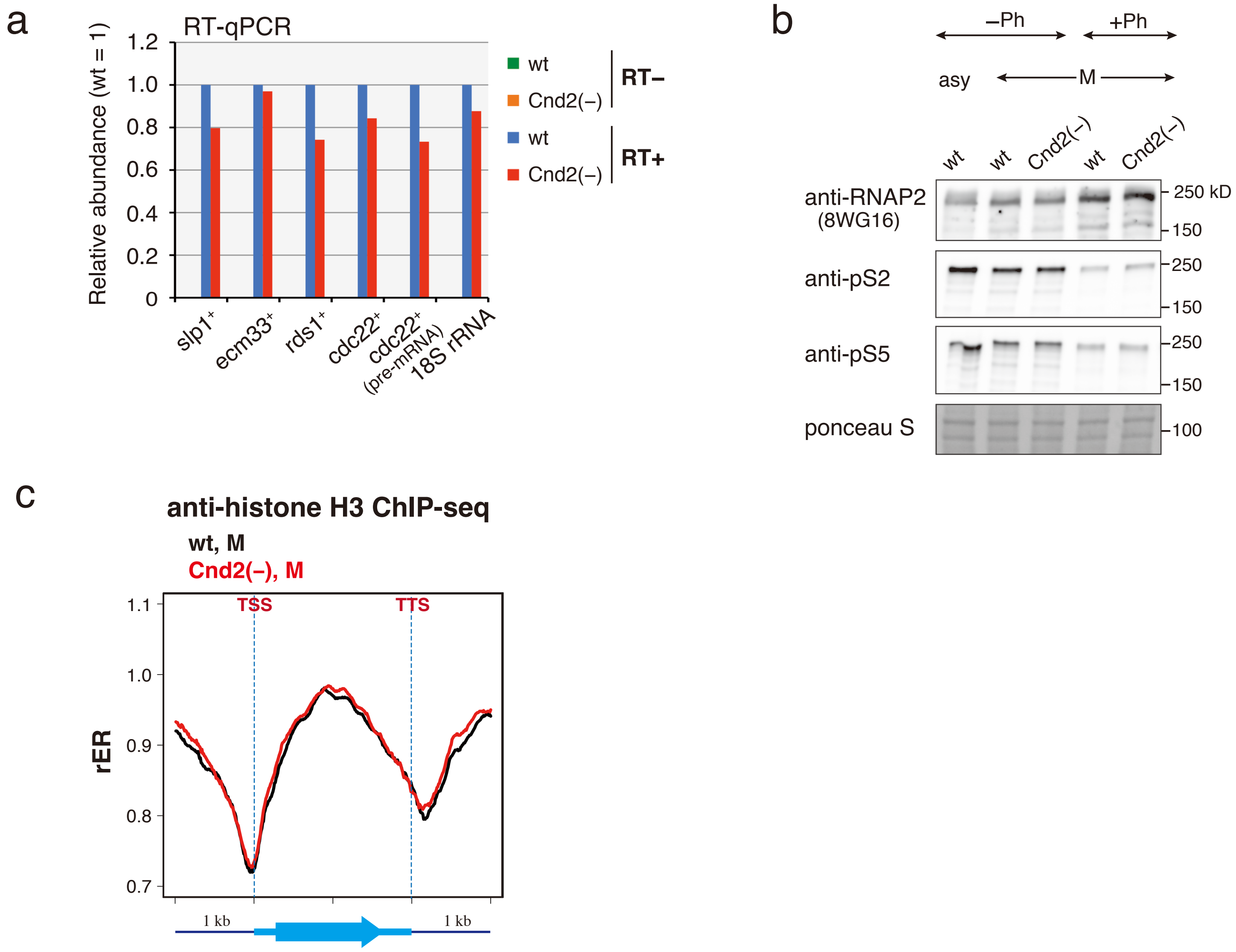

Supplementary Figure 5

Condensin depletion affects neither mitotic gene expression nor nucleosome distribution on active genes. (**a**) Condensin has little impact on gene transcription. RNA levels in wt and condensin-depleted (Cnd2(−)) cells were measured by RT-qPCR using random primers. mRNA and rRNA levels were comparable between the two strains. One primer set was designed to detect *cdc22*$^{+}$ pre-mRNA to monitor nascent RNA products. No detectable PCR products were evident in the absence of RT, ruling out the possibility of genomic DNA contamination. (**b**) Cnd2 depletion has little effect on levels of active RNAP2 (phosphorylated at Ser2 or Ser5 in the CTD repeat region, quantified by immunoblotting with anti-pS2 or anti-pS5, respectively). Asy, asynchronous cells; M, prometaphase-arrested cells. Upon treatment with 1,10-phenanthroline (Ph) for 30 min, the amount of active (i.e., phosphorylated) RNAP2 was decreased, confirming the specificity of the antibodies against active RNAP2. (**c**) Metagene ChIP-seq profiles of histone H3 in wt (black) and Cnd2-depleted (red) cells arrested in mitosis. Profiles were averaged over the condensin-bound genes (top 10% of ranked genes in Fig. 1d). The profile is from 1 kb upstream of the TSS to 1 kb downstream of the TTS, and gene lengths are scaled to the same size. The nucleosome density profile was indistinguishable between these two conditions. All the experiments in this figure were performed once.

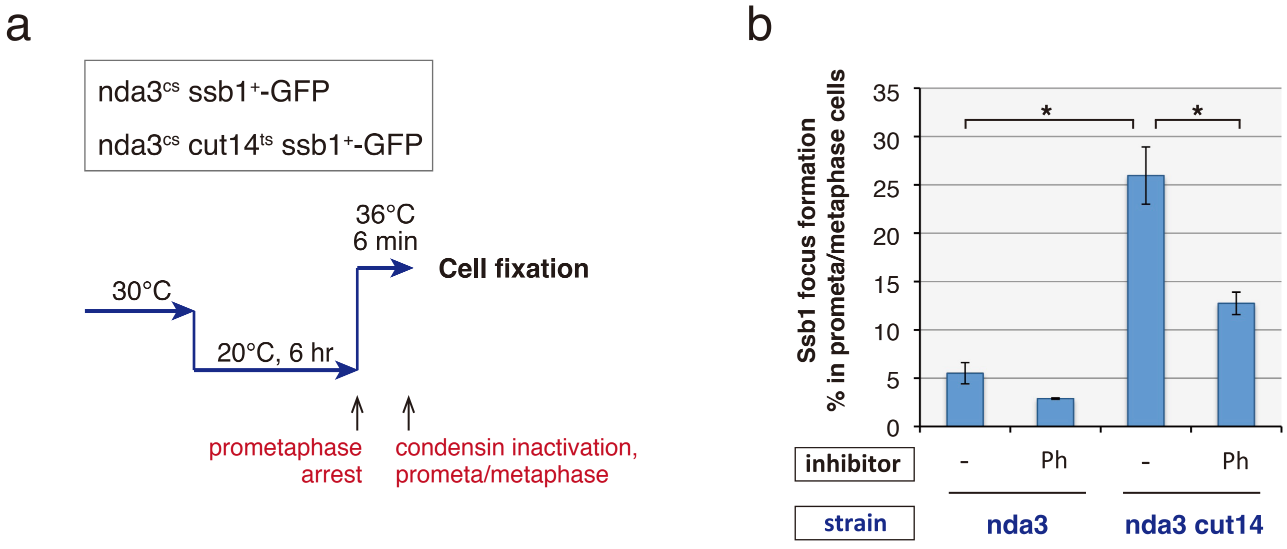

Supplementary Figure 6

Supplementary data for Figure 5 (opposing roles of transcription and condensin in DNA unwinding). (**a**) Experimental procedure. *nda3* or *nda3 cut14* cells expressing GFP-tagged Ssb1 were arrested at prometaphase by cultivating at 20 ° C for 6 h, transferred to 36 ° C for 6 min to inactivate condensin, and then fixed. Few cells showed divided/stretched nuclei (data not shown), indicating that the cells were in either prometaphase or metaphase. (**b**) Frequency of Ssb1-GFP focus formation in prometa/metaphase cells. *nda3 cut14* mutant cells showed increased Ssb1 focus formation compared with *nda3* cells, and 1,10-phenanthroline (Ph) treatment attenuated this effect (*$p < 0.05$; Welch' s t-test, one-tailed). Error bars represent s.e.m. ($n = 3$, independent experiments). The result indicates that mitotic function of condensin is required to repress Ssb1 accumulation on chromatin.

a

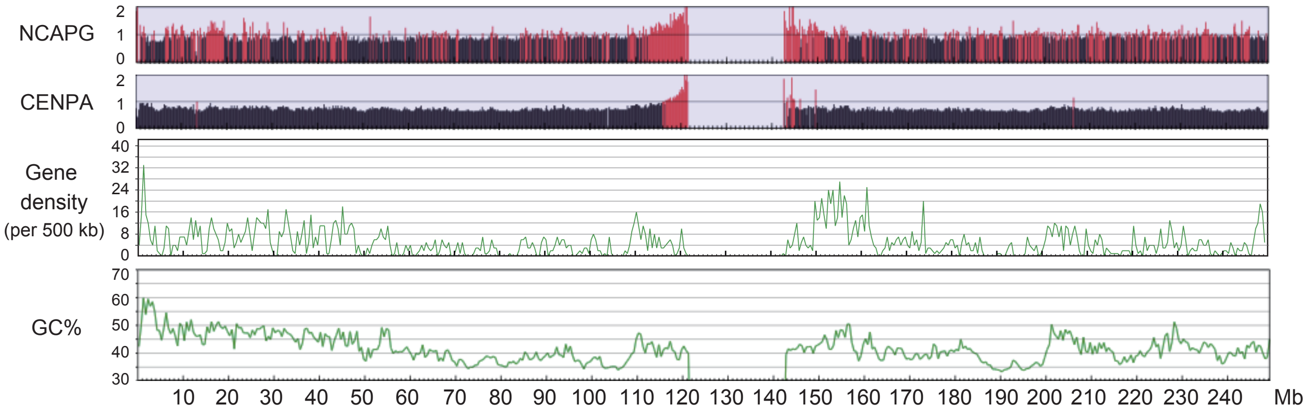

b

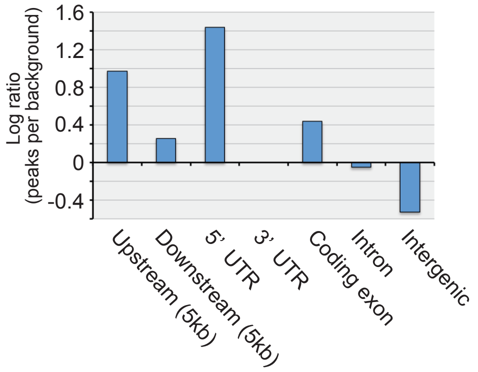

Supplementary Figure 7

Supplementary data (1) for Figure 6 (binding of human condensin I to the TSS of active genes transcribed by RNAP2 and RNAP3). (**a**) Chromosome-wide distribution of NCAPG (condensin I) and GFP-tagged centromere-specific histone H3 variant CENPA as revealed by ChIP-seq, along with gene density and GC content of chromosome 1. Red indicates regions with ChIP enrichment. The region with no ChIP-seq signal at ~130 Mb corresponds to the centromere, the entire sequence of which is not available in the human genome. (**b**) Classification of condensin I binding sites in non-repetitive regions of the human genome. The y axis is the ratio of the observed peak number to the expected value under the assumption of random distribution on a $\log_{10}$ scale. Condensin I binding sites are enriched in TSS-proximal regions (i.e., upstream and 5′ untranslated region [UTR]).

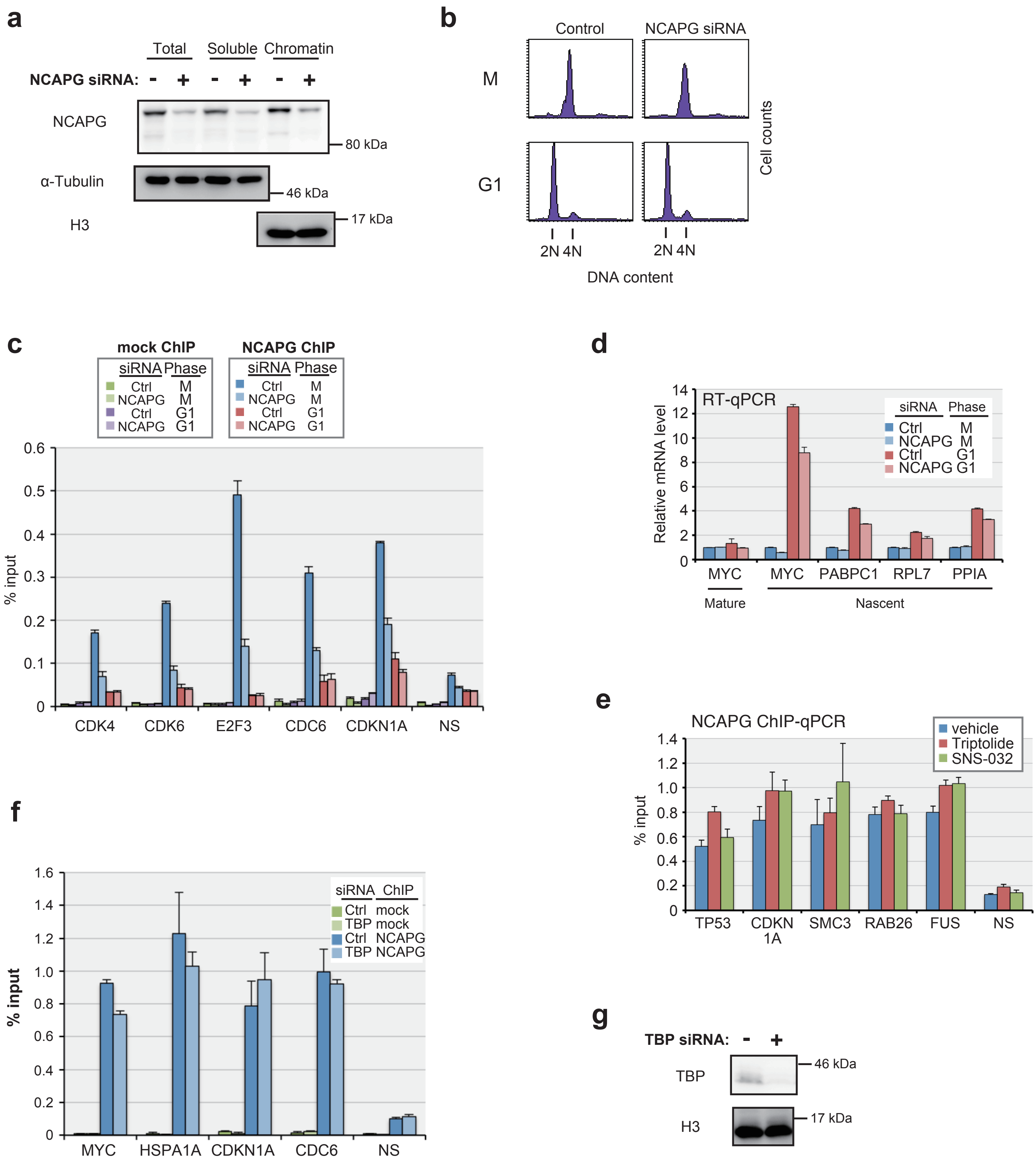

Supplementary Figure 8

Supplementary data (2) for Figure 6 (binding of human condensin I to the TSS of active genes transcribed by RNAP2 and RNAP3). (**a**) Verification of NCAPG subunit siRNA by immunoblotting. Total cell lysates from prometaphase-arrested cells were fractionated into soluble and chromatin-bound fractions and analysed. α-Tubulin and histone H3 were used as controls for soluble and chromatin-bound proteins, respectively. (**b**) Verification of cell cycle synchronisation in prometaphase and G1 by FACS analysis. (**c**) ChIP-qPCR confirmation of condensin I peaks. Mock ChIP, ChIP using nonspecific IgG beads; NCAPG ChIP, ChIP using anti-NCAPG beads. M, prometaphase-arrested cells; G1, cells synchronised in G1. Ctrl, vehicle treated control; NCAPG, siRNA knockdown of NCAPG subunit. NS represents a non-condensin-binding site (as a control). (**d**) RT-qPCR analysis of several condensin I–bound genes. The mature primer pair detects spliced mRNA, whereas the nascent primer pairs detect pre-spliced, nascent RNA products. M, cells arrested in prometaphase; G1, cells synchronised in G1 phase. Ctrl, vehicle treated control; NCAPG, siRNA knockdown of NCAPG subunit. The y axis is the relative RNA amount, where the amount in M phase cells without siRNA (Ctrl) was set to 1. (**e**) Effect of transcription inhibitors on condensin I binding. ChIP-qPCR of NCAPG in prometaphase cells treated with triptolide (1 μM) or SNS-032 (200 nM) for 3.5 h. Each inhibits RNAP2 transcription in human cells. (**f**) Effect of TBP knockdown on condensin I binding. ChIP-qPCR of NCAPG in cells treated with TBP siRNA. Ctrl, vehicle treated control; mock, ChIP using nonspecific IgG beads. Error bars represent s.d. (n = 3, technical replicates in qPCR). This experiment was performed once. (**g**) Confirmation of TBP depletion by immunoblotting of the cell lysate.

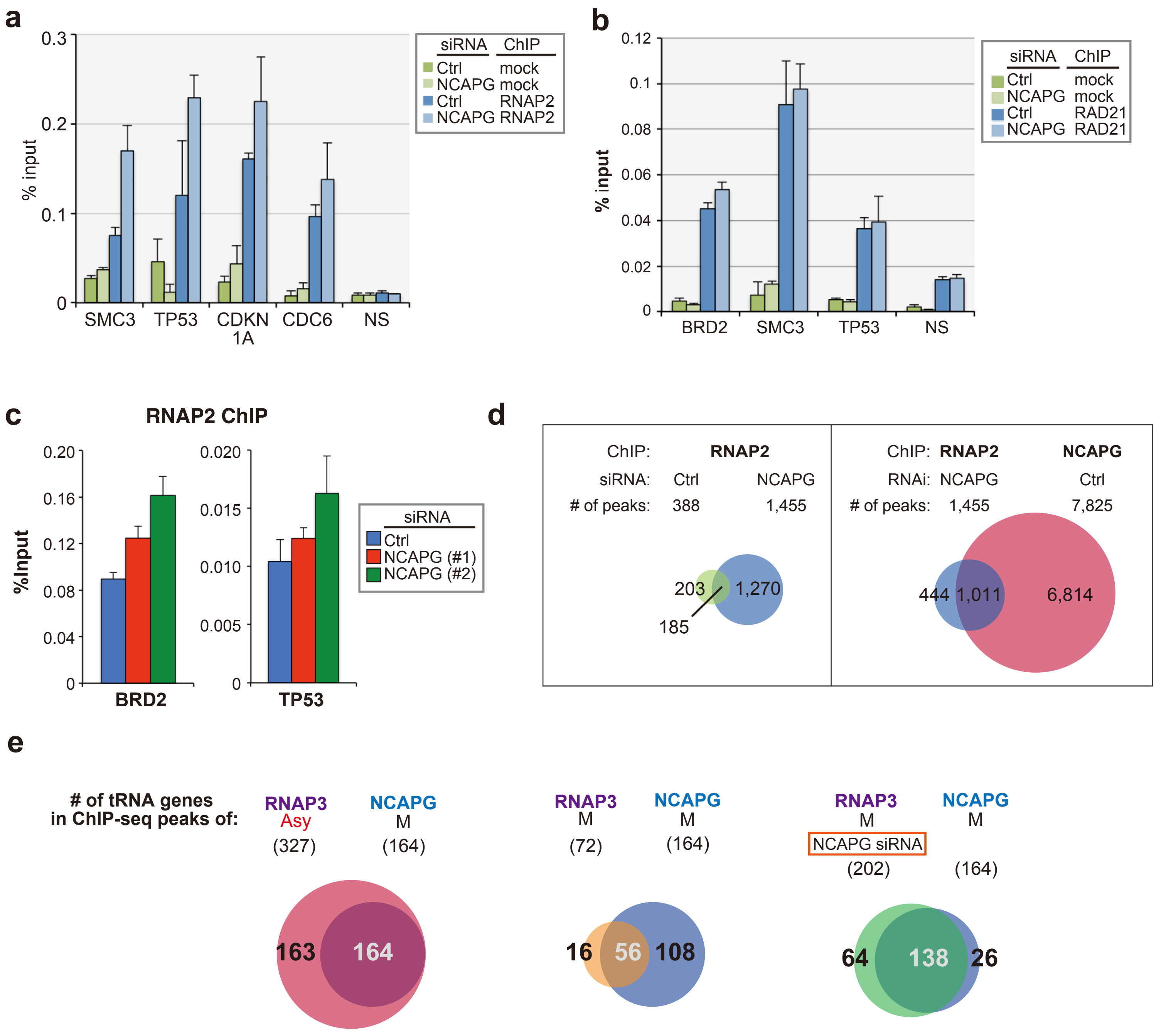

Supplementary Figure 9

Supplementary data for Figure 7 (recognition of ssDNA and expulsion of RNAPs from TSSs by human condensin I). (**a,b**) ChIP-qPCR analysis of RNAP2 (a) and cohesin (b) binding at TSSs in prometaphase-arrested cells. RNAP2, ChIP using anti-RNAP2 beads; RAD21, ChIP using anti-RAD21 beads; mock, ChIP using nonspecific IgG beads. NCAPG, siRNA knockdown of NCAPG subunit; Ctrl, vehicle treated control. NS represents a non-condensin-binding site (as a control). Condensin I depletion increased chromosomal binding of RNAP2, but not cohesin, at the condensin binding sites. Experiment in (b) was performed once. (**c**) ChIP-qPCR analysis of RNAP2 using a different NCAPG siRNA. Ctrl, vehicle treated control. NCAPG (#1), siRNA knockdown of NCAPG subunit by the same siRNA used in the other experiments. NCAPG (#2), siRNA knockdown of NCAPG subunit by another siRNA oligo. Experiment in (c) was performed once. (**d**) Venn diagrams of peaks detected by ChIP-seq. RNAP2 peaks in control cells showed no significant correlation with RNAP2 peaks in NCAPG–depleted cells (left). In contrast, the majority of RNAP2 peaks in NCAPG–depleted cells overlapped with those of NCAPG in control cells (right). (**e**) Venn diagrams of tRNA genes overlapping with the indicated ChIP-seq peaks. tRNA genes with NCAPG binding were expressed (associated with RNAP3) in asynchronous cells. About one-third of tRNA genes with NCAPG binding were also associated with RNAP3 in normal mitotic cells. tRNA genes with NCAPG binding in normal mitotic cells showed significant overlap with tRNA genes with RNAP3 binding in NCAPG–depleted mitotic cells. Error bars in this figure represent s.d. (n = 3, technical replicates in qPCR).

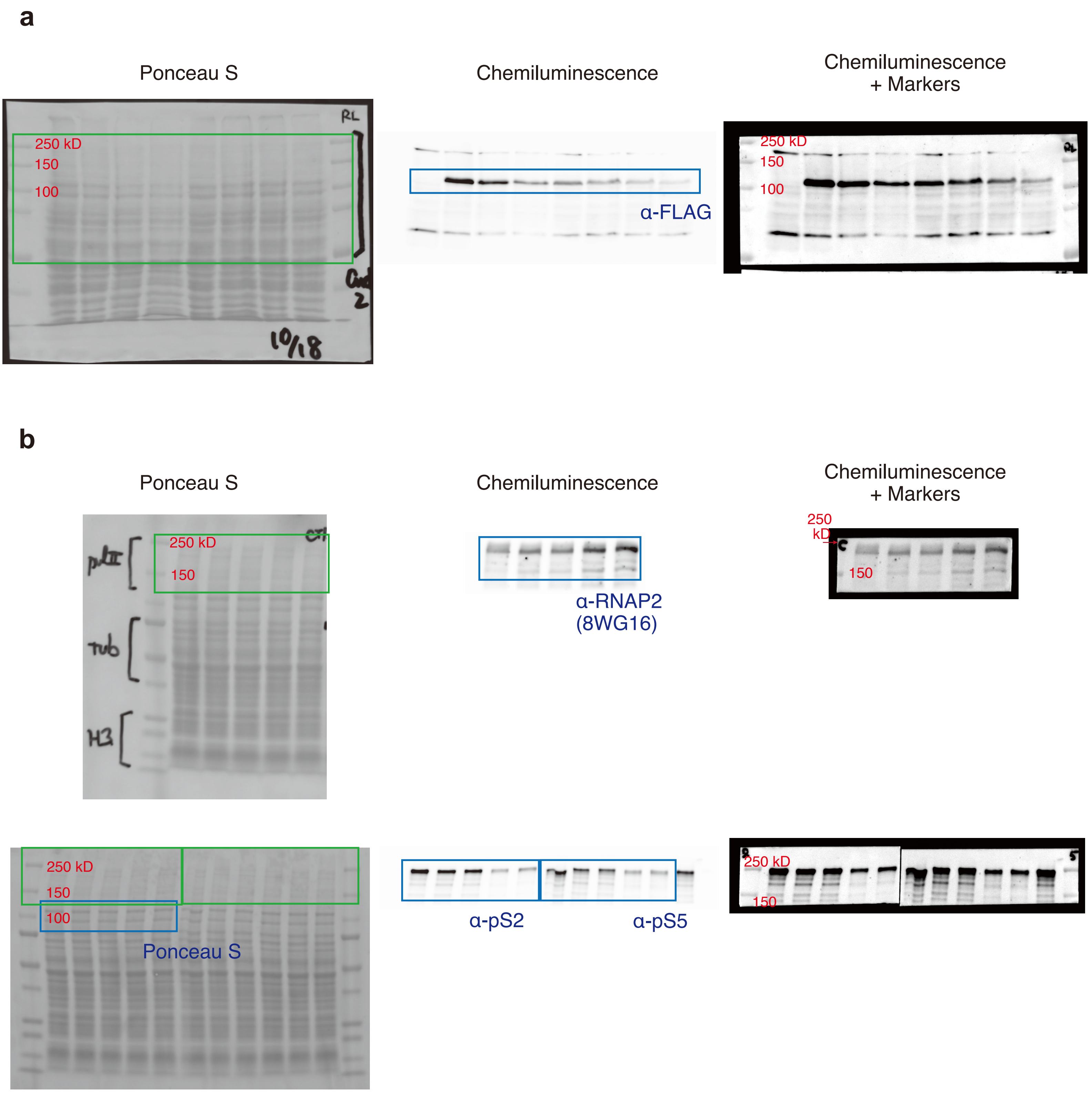

Supplementary Figure 10
Images of the uncropped immunoblots with molecular weight markers of the blot data shown in Supplementary Figures 4 and 5. (**a, b**) Ponceau S, images of ponceau S-stained membranes. The areas indicated by green boxes were cut out and subjected to immunodetection. Chemiluminescence, images of chemiluminescence immunoblots. Chemiluminescence + Markers, the chemiluminescence images overlaid with images of prestained molecular weight size markers on the same membranes. The areas indicated by blue boxes were cropped and used for Supplementary Figure 4b (a) and Supplementary Figure 5b (b). Sizes of molecular weight markers are shown in red.

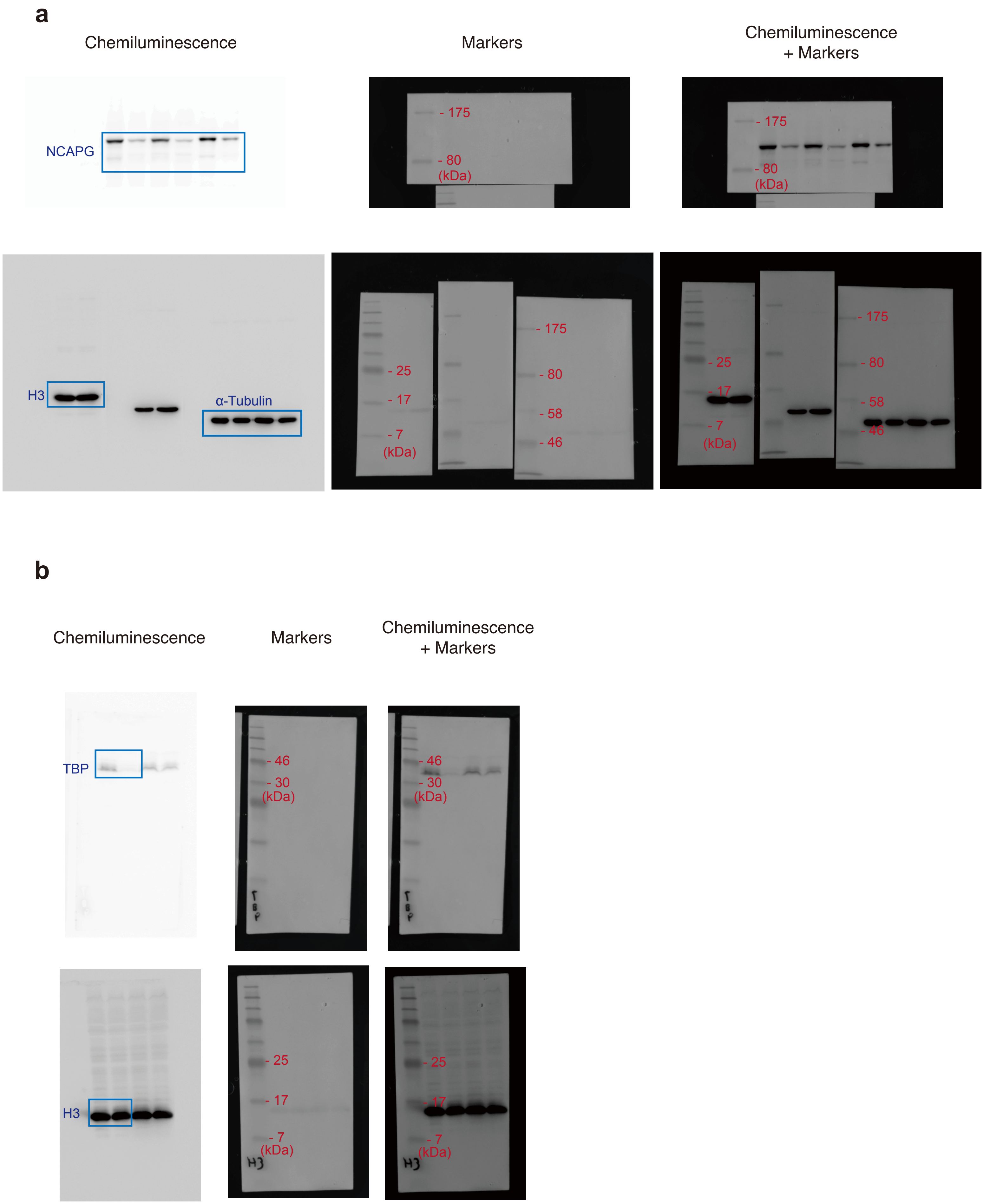

Supplementary Figure 11

Images of the uncropped immunoblots with molecular weight markers of the blot data shown in Supplementary Figures 8. (**a, b**) Chemiluminescence, images of chemiluminescence immunoblots. Markers, positions of prestained molecular weight size markers on the same membranes, revealed by white light epi-illumination. Chemiluminescence + Markers, the chemiluminescence images overlaid with the marker images. The areas indicated by blue boxes were cropped and used for Supplementary Figure 8a (a) and Supplementary Figure 8g (b). Sizes of molecular weight markers are shown in red.

Supplementary Table 1

A list of genomic positions where fission yeast condensin (Cut14-PK) is strongly enriched.

Peak ID	Chr.	Start	End	Peak summit	Peak width	Max rER	Associated feature(s)
1	I	1568800	1570949	1569565	2149	166	ecm33
2	II	1684190	1685019	1684645	829	141.9	ran1
3	II	1307970	1309519	1308885	1549	98.7	sol1, snoU14
4	I	1666740	1668739	1667725	1999	61.4	rds1
5	I	3765100	3777179	3770895	12079	57	cen1
6	II	3515090	3517339	3516775	2249	56	exg1
7	I	4877780	4880199	4878375	2419	54.7	gas1
8	II	1618880	1629539	1625075	10659	41.9	cen2
9	II	3958010	3958909	3958505	899	40.5	grx4, snu32
10	II	2021280	2022749	2021945	1469	38.8	SPNCRNA.1482
11	I	4077760	4079449	4078215	1689	38.4	adg2
12	I	1000210	1003129	1002625	2919	35.2	eng1
13	I	4007620	4009549	4008845	1929	29.8	SPAC27E2.13, SPAC27E2.11c, prl53
14	I	3288100	3288799	3288325	699	28.5	SPAPB1E7.04c
15	III	2448100	2448569	2448105	469	27.1	intergenic in rRNA gene repeat
16	III	1099840	1100729	1100275	889	27	cen3
17	I	4228150	4230739	4230175	2589	26.7	cdc22
18	II	1629560	1630409	1629955	849	25.5	cen2
19	III	4720	5179	5165	459	25.3	intergenic in rRNA gene repeat
20	III	15590	16049	16035	459	25.3	intergenic in rRNA gene repeat
21	II	1689180	1689949	1689255	769	24.6	fba1
22	II	1646000	1646339	1646325	339	22.8	tRNAs in otr of cen2
23	II	1600670	1601009	1600995	339	22.7	tRNAs in otr of cen2
24	II	1618020	1618869	1618465	849	22.2	cen2
25	II	1645250	1645999	1645625	749	22.2	tRNAs in otr of cen2
26	II	1599920	1600659	1600285	739	21.8	tRNAs in otr of cen2
27	I	1924560	1925459	1925135	899	21.4	tos4, bos1
28	III	5600	5999	5605	399	18.7	intergenic in rRNA gene repeat
29	III	16470	16869	16475	399	18.7	intergenic in rRNA gene repeat
30	III	2447270	2447659	2447645	389	18.1	intergenic in rRNA gene repeat
31	II	2415810	2416749	2416255	939	16.9	ape2, SPNCRNA.1511
32	II	625410	626399	625895	989	16.1	ef1a-c
33	I	3710170	3711209	3710685	1039	15.7	ppk3, SPNCRNA.229, SPNCRNA.929, SPATRNAVAL.04
34	III	1097350	1098099	1097715	749	15.3	cen3
35	III	2216960	2217619	2217285	659	15.2	SPCC1919.05, SPNCRNA.584
36	I	4140750	4141579	4141105	829	13.9	tif51
37	I	3871300	3871829	3871465	529	13.5	SPAC1071.09c, pma1
38	II	1971770	1972669	1972265	899	12.8	SPBC1E8.05
39	II	2181250	2181989	2181605	739	12.3	prs2402, cnt5
40	I	5306310	5307139	5306695	829	12.1	gas5
41	II	1601960	1602849	1602265	889	12	tRNAs in otr of cen2
42	II	1742220	1743039	1742595	819	11.8	SPCNRNA.1459
43	II	4535730	4536189	4535735	459	11.8	
44	I	3897140	3897599	3897285	459	11.7	dlc2
45	III	1413410	1414189	1413795	779	11.4	htb1, SPCC622.10c
46	III	1105460	1106079	1105765	619	11.3	cen3
47	III	1092930	1093539	1093235	609	11.1	cen3
48	I	3851090	3851889	3851435	799	11.1	SPAC4H3.12c, SPNCRNA.942, snr62
49	II	338590	339429	339165	839	11	sme2
50	II	478720	479279	479095	559	10.8	cdt1, utp15
51	I	527190	528149	527985	959	10.7	psf3
52	III	2442830	2443239	2442835	409	10.6	intergenic in rRNA gene repeat
53	I	997420	998259	997905	839	10.5	SPNCRNA.163
54	III	10030	10439	10425	409	10.4	intergenic in rRNA gene repeat
55	III	20900	21309	21295	409	10.4	intergenic in rRNA gene repeat
56	II	3020330	3020919	3020675	589	10.4	snu1
57	I	4748200	4748959	4748505	759	9.9	mid2
58	II	2303870	2305029	2304485	1159	9.8	sad1
59	I	994690	995659	995645	969	9.5	slp1
60	III	2448680	2449069	2448685	389	9.2	intergenic in rRNA gene repeat
61	III	4220	4609	4595	389	9.1	intergenic in rRNA gene repeat
62	III	15090	15479	15465	389	9.1	intergenic in rRNA gene repeat
63	I	958710	959279	958895	569	9	SPAC222.08c, snu2
64	I	4431560	4432099	4431715	539	8.6	sfp1
65	I	3238880	3239419	3239045	539	8.6	ace2
66	I	4424340	4425009	4424745	669	7.9	srp2
67	I	338930	339639	339255	709	7.6	taf9
68	II	2215570	2216119	2215755	549	7.6	cdc18
69	II	2681670	2682369	2682325	699	7.2	cfh4
70	II	1643770	1644459	1644145	689	6.9	tRNAs in otr of cen2
71	I	960340	960849	960565	509	6.5	seb1
72	II	887290	887799	887415	509	6.4	tim16, pmp3
73	II	1598640	1599119	1598805	479	6.1	tRNAs in otr of cen2
74	I	3395260	3395699	3395525	439	6	SPAC959.05c, SPNCRNA.906
75	III	814740	815209	814865	469	6	SPCC1393.08
76	I	1923580	1924549	1924225	969	5.5	tos4

Supplementary Table 2

Schizosaccharomyces pombe strains used in this study.

Relevant figure	Strain	Genotype
Fig. 1 abd	ST207	h^- *ura4 nda3-KM311 cut14*$^+$-9PK:*ura4*$^+$
Fig. 1 c	FY11487*	h^- *nda3-KM311*
	ST207	h^- *ura4 nda3-KM311 cut14*$^+$-9PK:*ura4*$^+$
	ST529	h^- *leu1 nda3-KM311 cut14-208*-9PK:*kanMX*
Fig. 2 a	FY11487*	h^- *nda3-KM311*
	ST207	h^- *ura4 nda3-KM311 cut14*$^+$-9PK:*ura4*$^+$
	ST611	h^- *ura4 nda3-KM311 rpb5*$^+$-9PK:*ura4*$^+$
Fig. 2 bc	ST207	h^- *ura4 nda3-KM311 cut14*$^+$-9PK:*ura4*$^+$
Fig. 3 a	FY11487*	h^- *nda3-KM311*
	FY7946*	h^- *leu1 nda3-KM311 cut14-208*
Fig. 3 b	ST553	*cut3* revertant #4 (h^- *leu1 cut3-477 med6 (L63X)*)
	ST563	*cut3* revertant #11 (h^- *leu1 cut3-477 med6 (D65fs)*)
	ST568	*cut3* revertant #31 (h^- *leu1 cut3-477 med6 (N95T)*)
Fig. 3 cd	ST001	972 (h^-)
	ST651	h^- *med6*::*kanMX*
	FY8026*	h^- *leu1 cut3-477*
	ST652	h^- *leu1 cut3-477 med6*::*kanMX*
	FY8027*	h^- *leu1 cut14-208*
	ST653	h^- *leu1 cut14-208 med6*::*kanMX*
Fig. 3 e	FY11487*	h^- *nda3-KM311*
	ST654	h^- *nda3-KM311 med6*::*kanMX*
Fig. 4 a	ST207	h^- *ura4 nda3-KM311 cut14*$^+$-9PK:*ura4*$^+$
	FY19976*	h^- *rad21*$^+$-GFP:*kanMX*
Fig. 4 b	ST207	h^- *ura4 nda3-KM311 cut14*$^+$-9PK:*ura4*$^+$
Fig. 5 a	ST207	h^- *ura4 nda3-KM311 cut14*$^+$-9PK:*ura4*$^+$
	ST610	h^- *ura4 nda3-KM311 ssb1*$^+$-9PK:*ura4*$^+$
Fig. 5 b	ST610	h^- *ura4 nda3-KM311 ssb1*$^+$-9PK:*ura4*$^+$
	ST617	h^- *ura4 nda3-KM311 ssb1*$^+$-9PK:*ura4*$^+$ *cnd2*::*P*$_{rep81}$*-3FLAG-cnd2*$^+$:*kanMX*
Fig. 5 cd	ST655	h^- *ssb1*$^+$-GFP:*kanMX locus_C*::*P*$_{adh15}$-mCherry-*atb2*$^+$:*hphMX*
	ST656	h^- *leu1 cut14-208 ssb1*$^+$-GFP:*kanMX locus_C*::*P*$_{adh15}$-mCherry-*atb2*$^+$:*hphMX*
	ST658	h^- *leu1 cut14-208 med6*::*natMX ssb1*$^+$-GFP:*kanMX locus_C*::*P*$_{adh15}$-mCherry-*atb2*$^+$:*hphMX*
	ST660	h^- *leu1 top2-342 ssb1*$^+$-GFP:*kanMX locus_C*::*P*$_{adh15}$-mCherry-*atb2*$^+$:*hphMX*
Supp. Fig. 1 af	ST207	h^- *ura4 nda3-KM311 cut14*$^+$-9PK:*ura4*$^+$
Supp. Fig. 1 bd	FY11487*	h^- *nda3-KM311*
	ST207	h^- *ura4 nda3-KM311 cut14*$^+$-9PK:*ura4*$^+$
Supp. Fig. 1 c	ST643	h^+ *leu1 nda3-KM311 aur1*:: *P*$_{adh21}$-NLS-GFP-9PK:*aur1*^{R}
	ST207	h^- *ura4 nda3-KM311 cut14*$^+$-9PK:*ura4*$^+$
Supp. Fig. 1 e	PH470†	h^- *ade6 nda3-KM311 locus_Z*::*P*$_{adh21}$-*cnd2*$^+$-GFP:*natMX*
	PH471†	h^- *ade6 nda3-KM311 locus_Z*::*P*$_{adh21}$-*cnd2(S5,41,52A)*-GFP:*natMX*
Supp. Fig. 2 a	ST001	972 (h^-)
	FY11487*	h^- *nda3-KM311*
Supp. Fig. 2 b	ST001	972 (h^-)
Supp. Fig. 2 cd	ST207	h^- *ura4 nda3-KM311 cut14*$^+$-9PK:*ura4*$^+$
Supp. Fig. 2 ef	ST591	h^- *ura4 nda3-KM311 cut14*$^+$-9PK:*ura4*$^+$ *aur1*::*P*$_{slp1}$-GFP:*aur1*^{R}
	ST593	h^- *ura4 nda3-KM311 cut14*$^+$-9PK:*ura4*$^+$ *aur1*::*P*$_{meu13}$-GFP:*aur1*^{R}
	ST594	h^- *ura4 nda3-KM311 cut14*$^+$-9PK:*ura4*$^+$ *aur1*::*P*$_{slp1(TATA\text{-}less)}$-GFP:*aur1*^{R}
Supp. Fig. 3 a	ST001	972 (h^-)
	FY8026*	h^- *leu1 cut3-477*
	FY8027*	h^- *leu1 cut14-208*
	FY11353*	h^- *leu1 top2-342*
Supp. Fig. 3 b	FY11487*	h^- *nda3-KM311*
	ST654	h^- *nda3-KM311 med6*::*kanMX*
Supp. Fig. 4 b	ST001	972 (h^-)
	ST634	h^- *cnd2*::*P*$_{cnd2}$*-3FLAG-cnd2*$^+$:*kanMX*
	ST635	h^- *cnd2*::*P*$_{rep81}$*-3FLAG-cnd2*$^+$:*kanMX*
Supp. Fig. 4 de, 5 ac	FY11487*	h^- *nda3-KM311*
	ST608	h^- *nda3-KM311 cnd2*::*P*$_{rep81}$*-3FLAG-cnd2*$^+$:*kanMX*
Supp. Fig. 5 b	ST001	972 (h^-)
	FY11487*	h^- *nda3-KM311*
	ST608	h^- *nda3-KM311 cnd2*::*P*$_{rep81}$*-3FLAG-cnd2*$^+$:*kanMX*
Supp. Fig. 6 b	ST601	h^- *nda3-KM311 ssb1*$^+$-GFP:*kanMX locus_C*::*P*$_{adh15}$-mCherry-*atb2*$^+$:*hphMX*
	ST602	h^- *nda3-KM311 cut14-208 ssb1*$^+$-GFP:*kanMX locus_C*::*P*$_{adh15}$-mCherry-*atb2*$^+$:*hphMX*

*Obtained from National BioResource Project (NBRP) − Yeast.
†Created and described previously (ref 1).

Supplementary Table 3

Primer pairs used in ChIP-qPCR and RT-qPCR analyses.

	Target site	Forward sequece (5′–3′)	Reverse sequence (5′–3′)	Chromosome position
S. pombe				
ChIP-qPCR				
	cnt*	GCAATATAATTCATAGTGTGGATACTTTTAGTC	TGGTTGTTGTTTTATGCGTGTACTCCAT	chr I: 3,770,739 - 3,770,893; chr III: 1,100,123 - 1,100,277
	ecm33†	CTAGTAGCAGAGACAGTGGAAGAC	GTGCTCTCACTGTCGAGACTAAG	chr I: 1,569,479 - 1,570,320
	slp1†	TTCACTAGAGTGTCCCTGAAGAGTC	CTCCTGGAATCGTCACGTTCTG	chr I: 996,025 - 996,136
	rds1†	GCAAAACTTCCCTGCCTTACATTTGG	GCGGGATACATGTAGGTACCGTTAG	chr I: 1,668,808 - 1,668,909
	ppk8	GTCCGGCATTCTACGGTATTTGC	CGACAATTCCCAATTTAGTAATGTTTATATAGC	chr I: 749,415 - 749,594
	meu13	GATATCGGTTTCATTCAATTTGTGAAGTG	GTACTCATAACGATGGGTTGAATTGC	chr I: 977,301 - 977,524
	rDNA*	GAAGAAAAAGTCGAGCGAGTCGAT	AAGTGCATTACCCTTACCTTTTATCTCTTC	chr III: 5,744 - 5,873; chr III: 16,614 - 16,743; chr III: 2,447,390 - 2,447,520
	psf3	CACATATAGATTAGCGTGCTATTCTG	CCACAGTATTGAGAGTATCACATAATGC	chr I: 527,945 - 528,070
	met26	GGCTAAGGAAGCTGGTATCATCAC	AAGCTTGGGCAAGAGTTCAATAGG	chr I: 1,479,085 - 1,479,202
	SPAC13C5.05c	CTTATAAGTATTGCTCCATGCACTAG	GAGCAATTACAAAGCTATTCCGTTTTG	chr I: 431,814 - 431,942
	ura1	GAAAGGTCAGGTCAACGAGCATTG	AGTCGAACCTGTTGGATATACTAACAC	chr I: 731,975 - 732,114
	SPAC27F1.05c	TGTCATACATAGTAAATTAACAACTCCGTTT	TCTAGTTTCAGCTTTTATATGCTCCT	chr I: 4,323,394 - 4,323,564
	cox3_mit	GGTTTAGAGATATGTCAACTGAAGCT	GAAGAAAGCCCAGAAAATAGAAGC	chr MT: 9,146 - 9,282
	chf1	CATACCAAAATCAAAAGTGGTGGTGGTG	GAATACTTTGGCGTTGCATAGAGC	chr I: 218,442 - 218,556
	pso2	GAAGGATAAAGGATTGGCATAGTAAAGC	GGGTTATACGAGCTTGTCTGTAGC	chr I: 1,156,960 - 1,157,068
	pss1	CTCCTTTTGCTCGTCAATTTCCATAG	TTTGAGGAAGGCACCGGTGGTC	chr I: 1,918,001 - 1,918,105
RT-qPCR				
	cdc22	GCTCGTGTTTCGAGGCTTTG	CGGGATACACACCAGAAATGACT	
	cdc22 (pre-mRNA)	TGACTGACTGTTGACATTGAATCG	CTCGAAACACGAGCAGTGATT	
	18S rRNA	AACCCAAAGACTTTGATTTCTCGTAAGG	AGACGATCAGATACCGTCGTAGTC	
	pre-rRNA	AACAAATTTTCGTTCAACACCTCATC	CCAAAGGCATGCCTGTTTGAG	
	GAPDH (human)	GGGAGTCCCTGCCACACTCAGT	AGCACAGGGTACTTTATTGATGGTACATG	
**Human**				
ChIP-qPCR				
	CDC6	AGGCGAAAGGCTCTGTGACTAC	CCACAAGCCCCTGAACAAAC	chr 17: 38,444,057 - 38,444,199
	CDKN1A	GCTGCGTTCACAGGTGTTTC	CTGTACTTGTAATCCCGCTCTCC	chr 6: 36,646,689 - 36,646,838
	CDK4	TGTGACCAGCTGCCAAAGAG	AGTCGAAGCACCTCCTGTCC	chr 12: 58,146,124 - 58,146,238
	CDK6	TTTACGAAGCCTCCATCGCTAC	TTCTCGGAGGAACCAGGAAG	chr 7: 92,463,095 - 92,463,174
	E2F3	CTCTTCTGCCAGCCAATCAAG	CCGCTACCTCCTTACTTCAGTCC	chr 6: 20,401,849 - 20,401,985
	FUS	TGGCCTCAAACGGTAGGTAAG	GGTCCCACTGAAAACGAAAAG	chr 16: 31,191,537 - 31,191,627
	RAB26	GGAAATCACAGAAAACACCAGAAAC	TGTGCAATCAAAAGGCCAAG	chr 16: 2,203,983 - 2,204,097
	SMC3	CAGACCTGAACTTGACTCCTCCTAC	CCCTCAGCCAAACAAAATGG	chr 10: 112,327,360 - 112,327,465
	TP53	GGTGGCTCTAGACTTTTGAGAAGC	GCGGATTACTTGCCCTTACTTG	chr 17: 7,590,789 - 7,590,934
	NS (Supp. Fig. 8 c)	GATGGAGACGCAACACTGC	GCTCACACACCTACGCTTCC	chr 13: 37,005,836 - 37,005,923
	NS (Supp. Fig. 8 ef, 9 ab)	ATCTTCACCTATGCCTGTGATTTG	TTCTTTCTTACCAGTCTCCGTGTG	chr 12: 7,942,273 - 7,942,372
	BRD2 (TSS)	TCTAGAACGAGCTGGAGGATTCTG	AGCGGATGGAGGTGGATTG	chr 6: 32,940,002 - 32,940,086
	BRD2 (gene body)	GAGGCAAGGGTCTTAAGTAAAGTGG	CAAGATGGCTGTAGGTGTAGGG	chr 6: 32,944,274 - 32,944,395
	BRD2 (TTS)	TTACTGCAGTGAGAAAAGGCAATAG	AAACACAGCAAAAGCCTACTGG	chr 6: 32,949,171 - 32,949,250
	RPL13 (TSS)	GCGACGTCAGTTCCTCCTTTC	GCAATGCACTCTGGGATGATAG	chr 16: 89,626,987 - 89,627,078
	RPL13 (gene body)	GTTAATGTAGCATCTTGGACTTTGG	AAGAAGCCCATTGAAGGAAGG	chr 16: 89,630,168 - 89,630,270
	RPL13 (TTS)	GTAGTTTCGACCCTGGAAGACG	CAAATGAAACTACGCCTGGATG	chr 16: 89,633,101 - 89,633,181
RT-qPCR				
	Myc	CCTGGTGCTCCATGAGGAGA	CTCCAGCAGAAGGTGATCCAGA	
	β-Actin	TGGCACCCAGCACAATGAA	CTAAGTCATAGTCCGAATAGAAGCA	

*Primer pairs that recognize multiple genomic sites. All amplicons are identical in sequence.
†Primer pairs also used for RT-qPCR.

Supplementary Table 4

Statistics for ChIP-seq, RNA-seq and whole-genome sequencing and mapping analyses.

	Sample description	Platform	Mapping tool (param)	Reference	Total number of reads	Number and percentage (in parentheses) of mapped reads	
S. pombe							
ChIP-seq							
	Cut14-PK, M (#1)	SOLiD 3	Bowtie 0.12.5 (-n2 -a)	972*h*- genome	15,093,827	10,976,893	(72.72)
	Cut14-PK, M, input (#1)	SOLiD 3	Bowtie 0.12.5 (-n2 -a)	972*h*- genome	17,870,654	12,195,442	(68.24)
	Cut14-PK, M (#2)	SOLiD 3	Bowtie 0.12.5 (-n2 -a)	972*h*- genome	21,626,672	9,345,751	(43.20)
	Cut14-PK, M, input (#2)	SOLiD 3	Bowtie 0.12.5 (-n2 -a)	972*h*- genome	23,191,107	12,649,797	(54.55)
	Cut14-PK, M, +Ph	SOLiD 5500	Bowtie 1.0.0 (-n2 -a)	972*h*- genome	5,176,265	4,217,607	(81.48)
	Cut14-PK, M, +Ph, input	SOLiD 5500	Bowtie 1.0.0 (-n2 -a)	972*h*- genome	6,032,260	5,160,548	(85.55)
	no_tag, M	SOLiD 5500	Bowtie 0.12.5 (-n2 -a)	972*h*- genome	17,302,708	7,724,772	(44.64)
	no_tag, M, input	SOLiD 5500	Bowtie 0.12.5 (-n2 -a)	972*h*- genome	12,429,625	9,648,032	(77.62)
	NLS-GFP-PK, M	HiSeq 2000	Bowtie 1.0.0 (-n2 -a)	972*h*- genome	6,880,537	5,588,890	(81.23)
	NLS-GFP-PK, M, input	HiSeq 2000	Bowtie 1.0.0 (-n2 -a)	972*h*- genome	7,279,549	7,211,943	(99.07)
	RNAP2, M	SOLiD 4	Bowtie 0.12.5 (-n2 -a)	972*h*- genome	22,623,891	15,293,783	(67.60)
	RNAP2, M, input	SOLiD 4	Bowtie 0.12.5 (-n2 -a)	972*h*- genome	21,961,496	13,626,041	(62.05)
	RNAP2 in *med6*Δ, M	HiSeq 2000	Bowtie 1.0.0 (-n2 -a)	972*h*- genome	8,377,501	8,325,764	(99.38)
	RNAP2 in *med6*Δ, M, input	HiSeq 2000	Bowtie 1.0.0 (-n2 -a)	972*h*- genome	7,916,214	7,854,152	(99.22)
	Rpb5-PK, M	HiSeq 2000	Bowtie 0.12.5 (-n2 -a)	972*h*- genome	9,882,330	9,809,614	(99.26)
	Rpb5-PK, M, input	HiSeq 2000	Bowtie 0.12.5 (-n2 -a)	972*h*- genome	9,689,100	9,596,404	(99.04)
	H3, M	HiSeq 2000	Bowtie 0.12.5 (-n2 -a)	972*h*- genome	7,523,375	7,478,607	(99.40)
	H3, M, input	HiSeq 2000	Bowtie 0.12.5 (-n2 -a)	972*h*- genome	7,557,186	7,403,444	(97.97)
	Ssb1-PK, M	HiSeq 2000	Bowtie 0.12.5 (-n2 -a)	972h- genome	8,802,105	8,541,026	(97.03)
	Ssb1-PK, M, input	HiSeq 2000	Bowtie 0.12.5 (-n2 -a)	972h- genome	9,001,604	8,845,764	(98.27)
RNA-seq							
	M	HiSeq 2000	Bowtie 0.12.5 (-n2 -a)	972*h*- genome	38,514,023	36,863,185	(95.71)
Genome sequencing							
	cut3	HiSeq 2000	Bowtie 2 2.1.0	972*h*- genome	84,011,924	83,647,192	(99.57)
	cut3 revertant #4	HiSeq 2000	Bowtie 2 2.1.0	972*h*- genome	87,886,448	87,494,603	(99.55)
	cut3 revertant #11	HiSeq 2000	Bowtie 2 2.1.0	972*h*- genome	72,394,477	72,106,308	(99.60)
	cut3 revertant #31	HiSeq 2000	Bowtie 2 2.1.0	972*h*- genome	60,532,222	60,284,501	(99.59)
**HeLa**							
ChIP-seq							
	NCAPG, M	HiSeq 2000	Bowtie 0.12.5 (-n3 -m1)	UCSC hg19	36,278,217	27,290,427	(75.23)
	NCAPG, M, input	HiSeq 2000	Bowtie 0.12.5 (-n3 -m1)	UCSC hg19	41,537,233	32,240,060	(77.62)
	RNAP2, M	HiSeq 2000	Bowtie 0.12.5 (-n3 -m1)	UCSC hg19	52,942,295	40,393,344	(76.30)
	RNAP2, M, input*	HiSeq 2000	Bowtie 0.12.5 (-n3 -m1)	UCSC hg19	213,329,370	170,144,879	(79.76)
	RNAP2, M, (NCAPG siRNA)	HiSeq 2000	Bowtie 0.12.5 (-n3 -m1)	UCSC hg19	53,570,759	41,164,206	(76.84)
	RNAP2, M, (NCAPG siRNA), input[†]	HiSeq 2000	Bowtie 0.12.5 (-n3 -m1)	UCSC hg19	62,539,714	47,908,147	(76.60)
	RNAP2, asy	HiSeq 2000	Bowtie 1.0.0 (-n3 -m1)	UCSC hg19	41,268,302	33,090,794	(80.18)
	RNAP2, asy, input[‡]	HiSeq 2000	Bowtie 1.0.0 (-n3 -m1)	UCSC hg19	138,806,083	108,543,661	(78.20)
	RNAP3, M	HiSeq 2000	Bowtie 0.12.5 (-n3 -m1)	UCSC hg19	46,524,340	35,694,248	(76.72)
	RNAP3, M, (NCAPG siRNA)	HiSeq 2000	Bowtie 0.12.5 (-n3 -m1)	UCSC hg19	33,643,225	25,975,867	(77.21)
	RNAP3, asy	HiSeq 2000	Bowtie 0.12.5 (-n3 -m1)	UCSC hg19	43,026,370	33,356,022	(77.52)
	GFP-CENPA, asy	HiSeq 2000	Bowtie 0.12.5 (-n3 -m1)	UCSC hg19	244,363,679	161,017,587	(65.89)
	GFP-CENPA, asy, input	HiSeq 2000	Bowtie 0.12.5 (-n3 -m1)	UCSC hg19	263,349,826	222,571,991	(84.52)

M, prometaphase cells; asy, asynchronous cells.
*Also used as input sample for HeLa "RNAP3, M"
†Also used as input sample for HeLa "RNAP3, M, (NCAPG siRNA)"
‡Also used as input sample for HeLa "RNAP3, asy"

Supplementary References

1. Tada, K., Susumu, H., Sakuno, T. & Watanabe, Y. Condensin association with histone H2A shapes mitotic chromosomes. *Nature* **474,** 477–483 (2011).
2. Nakazawa, N., Mehrotra, R., Ebe, M. & Yanagida, M. Condensin phosphorylated by the Aurora-B-like kinase Ark1 is continuously required until telophase in a mode distinct from Top2. *J. Cell Sci.* **124,** 1795–1807 (2011).
3. Saka, Y. *et al.* Fission yeast cut3 and cut14, members of a ubiquitous protein family, are required for chromosome condensation and segregation in mitosis. *EMBO J.* **13,** 4938–4952 (1994).
4. Sabatinos, S. A. & Forsburg, S. L. Molecular genetics of Schizosaccharomyces pombe. *Methods Enzymol.* **470,** 759–795 (2010).
5. Sutani, T. *et al.* Fission yeast condensin complex: essential roles of non-SMC subunits for condensation and Cdc2 phosphorylation of Cut3/SMC4. *Genes Dev.* **13,** 2271–2283 (1999).
